# Sleep Deprivation and Memory: Meta-Analytic Reviews of Studies on Sleep Deprivation Before and After Learning

**DOI:** 10.1037/bul0000348

**Published:** 2021-11

**Authors:** Chloe R. Newbury, Rebecca Crowley, Kathleen Rastle, Jakke Tamminen

**Affiliations:** 1 Department of Psychology, Royal Holloway, University of London

**Keywords:** sleep, sleep deprivation, learning, memory, meta-analysis

## Abstract

Research suggests that sleep deprivation both before and after encoding has a detrimental effect on memory for newly learned material. However, there is as yet no quantitative analyses of the size of these effects. We conducted two meta-analyses of studies published between 1970 and 2020 that investigated effects of total, acute sleep deprivation on memory (i.e., at least one full night of sleep deprivation): one for deprivation occurring before learning and one for deprivation occurring after learning. The impact of sleep deprivation after learning on memory was associated with Hedges’ *g* = 0.277, 95% CI [0.177, 0.377]. Whether testing took place immediately after deprivation or after recovery sleep moderated the effect, with significantly larger effects observed in immediate tests. Procedural memory tasks also showed significantly larger effects than declarative memory tasks. The impact of sleep deprivation before learning was associated with Hedges’ *g* = 0.621, 95% CI [0.473, 0.769]. Egger’s tests for funnel plot asymmetry suggested significant publication bias in both meta-analyses. Statistical power was very low in most of the analyzed studies. Highly powered, preregistered replications are needed to estimate the underlying effect sizes more precisely.

## Public Significance Statement

The health risks associated with lack of sleep are well known, but the consequences of missing one or more nights of sleep for learning and memory are less well appreciated. In two meta-analyses pooling studies across 5 decades of research, we found that total sleep deprivation before learning as well as after learning had a detrimental impact on memory for the newly learned materials. These data suggest sleep supports learning and memory in two ways: It prepares the brain for learning over the next day, and it helps strengthen new memories learned during the previous day.

There is a growing body of evidence suggesting a critical role of sleep in learning and memory (Diekelmann & Born, 2010). On the one hand, offline memory consolidation during sleep benefits both declarative and procedural memories acquired during preceding wake (Klinzing et al., 2019). On the other hand, memory encoding capacity has been argued to saturate gradually during wake, with sleep restoring this capacity (Cirelli & Tononi, in press; Tononi & Cirelli, 2012). These theoretical advances have been accompanied by practical societal concerns regarding the prevalence of poor sleep, especially for students (Twenge et al., 2017) and shift workers (Vidya et al., 2019). The proposed importance of sleep for memory processes has been supported by many studies showing detrimental effects of total sleep deprivation on the learning and retrieval of new information. Yet, the effect sizes associated with the total sleep deprivation impairment are variable, and some studies have failed to find significant effects altogether (e.g., Diekelmann et al., 2008). Therefore, we used a meta-analytic approach to estimate the effect size associated with the impact of sleep deprivation, both when the deprivation occurs before learning and when it occurs after learning.

## Impact of Total Sleep Deprivation After Learning and Potential Moderators of the Effect

The active systems consolidation theory suggests that sleep *after* learning strengthens new memories (e.g., Klinzing et al., 2019; Kumaran et al., 2016; McClelland et al., 1995). Information learned during wakefulness is initially encoded rapidly in the hippocampus, where memories are stored separately from existing memory stores. Repeated reactivation of these new memories, primarily during slow-wave sleep (SWS), supports the strengthening of memory representations and leads to the integration of these memories in the neocortex. Such neocortical representations are less liable to disruption and form interrelated semantic networks with existing memories, yielding memory representations that allow abstraction, generalization, and discovery of statistical patterns across discrete memories (Lewis & Durrant, 2011; Lewis et al., 2018; Stickgold & Walker, 2013). Notably, since the mechanisms outlined in this theory relate only to hippocampal-dependent memory consolidation (Inostroza & Born, 2013), these mechanisms may primarily support the consolidation of declarative (or explicit) memory.

Effects of sleep on nondeclarative memory have also been observed; for example, sleep enhances motor skills such as finger-tapping sequence learning (Korman et al., 2007; Walker et al., 2002; see King et al., 2017 for a review). However, this beneficial effect of sleep on procedural memories may be evident only when learning is intentional (explicit memory), rather than unintentional (implicit memory). Robertson et al. (2004) found that awareness of learning a finger-tapping task led to a sleep benefit, whereas improvements in implicit learning performance, when participants had little awareness of the task, were similar regardless of whether the retention interval contained sleep or wakefulness. Thus, there are suggestions that the mechanisms involved in the consolidation of hippocampal-dependent declarative memories may also facilitate the consolidation of some procedural tasks that rely on explicit learning and thus show some hippocampal-dependency (Schönauer et al., 2014; Walker et al., 2005).

However, this latter theory does not take into account observations that some procedural tasks that do not rely on explicit learning or an intact hippocampus still show superior performance after sleep. Stickgold et al. (2000) found that a period of sleep after learning a visual discrimination task benefited later performance, and Schönauer et al. (2015) found that improvements in performance on a mirror-tracing task were only observed after a period of offline consolidation. Recent studies in both animals (Sawangjit et al., 2018) and humans (Schapiro et al., 2019) have suggested that the hippocampus may be involved in the sleep-dependent consolidation of memories that do not rely on the hippocampus during encoding. For example, Schapiro et al. (2019) trained amnesic patients with hippocampal damage on the motor sequence task, a classic procedural memory task typically considered to be non-hippocampus-dependent. The patients were able to learn the task equally well compared to matched controls, suggesting that the hippocampus is not required for learning of the task. However, while the controls showed the expected overnight consolidation benefit, the patients did not, leading the authors to conclude that the hippocampus may be involved in the consolidation of procedural tasks that do not require it for initial learning.

As reviewed above, theories of memory consolidation predict that depriving participants of sleep after learning should impair memory for the information encoded before sleep deprivation, relative to control conditions where participants are allowed to sleep normally after learning. In our first meta-analysis, we analyze the current research into both declarative and procedural memories to estimate the size of this sleep deprivation after learning effect. We focus on the literature using manipulations of total sleep deprivation, as this is the strongest and most direct manipulation to test theories of sleep-associated memory consolidation. In doing so, we exclude from our analyses studies of sleep restriction. In standard sleep restriction studies, participants are allowed to sleep for a shorter duration than they would do otherwise, and the manipulation typically continues for multiple nights. This chronic sleep restriction can have a detrimental impact on learning and memory (e.g., Cousins et al., 2018) although not always (e.g., Voderholzer et al., 2011). In selective sleep restriction studies, participants are only deprived of the first half of the night, rich in SWS, or the second half of the night, rich in rapid eye movement (REM) sleep (e.g., Plihal & Born, 1997). These studies can reveal important information about the precise brain mechanisms underlying benefits of sleep on memory. There are two theoretically motivated reasons for excluding both types of sleep restriction from our meta-analyses. Standard sleep restriction studies still allow participants to sleep for several hours each night, and it may be sufficient for sleep-associated memory consolidation to occur. Thus, these manipulations do not provide a strong test of the relevant theories, and their inclusion might lead us to underestimate the effect size associated with sleep deprivation. Selective sleep restriction studies on the other hand are designed to address the more fine-grained question of which specific sleep stages are the most beneficial for memory. Different theories make different predictions in this regard: For example, the active systems consolidation theory emphasizes the role of SWS (Klinzing et al., 2019); the theory of Lewis et al. (2018) emphasizes REM (at least for learning involving creative thought); and the sequential theory of Giuditta (2014) proposes that an interaction between SWS and REM is key. However, all current theories make the common prediction that sleep should benefit memory more than wake. We do not attempt to adjudicate between the competing theories and therefore restrict our analysis to studies using total sleep deprivation where a clear prediction is made by all theories.

Where possible we use moderators to establish whether the effect size is modulated by variables that have been hypothesized to be important. For example, it is not clear whether sleep deprivation after learning impacts declarative and procedural memories similarly. If the neural and cognitive mechanisms associated with the consolidation of declarative and procedural memories differ, one may observe different effect sizes in studies targeting the two memory types. To test this hypothesis, we entered declarative versus procedural memory type as a moderator in our meta-analysis.

Some studies have tested the effects of sleep deprivation after one or more nights of recovery sleep while others have tested immediately after a night of sleep deprivation with no intervening recovery sleep. The primary reason for allowing recovery sleep before testing is that sleep deprivation has well-established impacts on attention (e.g., Lim & Dinges, 2008; Vargas et al., 2017) that may compromise performance in an immediate memory test and make it difficult to distinguish between effects of sleep deprivation due to fatigue and effects due to disruption to memory consolidation processes. Using recovery sleep to rule out potential effects of fatigue at test assumes that the first night of sleep following learning is of critical importance in memory consolidation and that consolidation may not occur in subsequent sleep periods or has a weaker impact after the first missed sleep opportunity. Although we are not aware of any studies that have explicitly tested this assumption, for example, by systematically manipulating the number of nights of sleep following learning, some support has been derived from studies such as that of Gais et al. (2007) who observed effects of one night of sleep deprivation after two recovery nights and even 6 months later.

Recent studies have cast doubt on the privileged status of the first night of sleep, however. Schönauer et al. (2015) found only a short-term cost of sleep deprivation after learning for hippocampal-dependent memories, with sleep deprivation after learning impairing retrieval of word pairs after one night of deprivation, but no difference in retrieval between the sleep and sleep deprivation conditions after two nights of recovery sleep. They suggested that for such hippocampal-dependent memories, the hippocampus may act as a temporary buffer, storing memories until the first sleep opportunity, even if that opportunity is delayed. Thus, sleep deprivation after learning would only have a detrimental effect on memory performance if there is no sleep opportunity before testing. In contrast, they found that procedural memories that do not rely on the hippocampus suffered a more long-term effect of sleep deprivation, supporting previous evidence that the first night of sleep is crucial for improving performance on procedural tasks (Stickgold et al., 2000). Therefore, for procedural memories, the loss of the first night of sleep may be critical. Given the mixed evidence in the literature on the impact of recovery sleep, we entered the presence or absence of recovery sleep as a moderator in the meta-analysis. If the first night of sleep after learning is of critical importance, on the one hand, we should see a sleep deprivation impairment both in studies that test immediately after deprivation and in studies that test after recovery sleep. If, on the other hand, consolidation processes can be delayed, we should see a sleep deprivation impairment only in studies that test immediately (i.e., they provide no opportunity for delayed consolidation). The latter scenario is also consistent with the possibility that the sleep deprivation impairment is due entirely to fatigue at test (although see Schönauer et al., 2015, for data suggesting fatigue does not impair memory recall).

Some studies of recognition memory have failed to find a beneficial effect of sleep on memory performance leading to a debate about whether sleep has no or only limited impact on recognition memory (e.g., Drosopoulos et al., 2005; Hu et al., 2006; Morgan et al., 2019; Rauchs et al., 2004; Stepan et al., 2017). The difference between recall and recognition tasks is particularly stark in the literature using the Deese–Roediger–McDermott (DRM) paradigm of false memory formation. Here, studies using recognition tasks have reported that sleep may reduce false memories (Fenn et al., 2009; Lo et al., 2014), whereas studies using recall tasks have reported that sleep may increase false memories (Diekelmann et al., 2010; McKeon et al., 2012; Pardilla-Delgado & Payne, 2017; Payne et al., 2009). Consequently, a recent meta-analysis of sleep studies using the DRM paradigm concluded that the impact of sleep on false memory is restricted to recall tasks (Newbury & Monaghan, 2019). This meta-analysis also found that the sleep benefit for studied words (i.e., veridical memory) was dramatically larger in recall tasks than in recognition tasks (*g* = 0.407 vs. *g* = 0.005). The discrepancy between recall and recognition tasks might be explained by dual-process accounts, which suggest that recognition memory has both an explicit recollection element as well as an implicit familiarity element (e.g., Jacoby, 1991; Yonelinas, 2002), and these two elements may rely on different neural structures, with only recollection depending on the hippocampus. Support for this account has come from findings that sleep only facilitates recognition memory based on recollection, not familiarity (e.g., Drosopoulos et al., 2005). On the other hand, studies that have directly compared memory for emotionally negative and neutral stimuli appear to suggest that sleep benefits recognition memory for emotional stimuli but not for neutral stimuli (Alger et al., 2018; Hu et al., 2006; Payne et al., 2008, 2015; see Kim & Payne, 2020 for a review) suggesting that sleep’s impact on recognition memory may be modulated by emotionality. Given the inconsistency in the existing literature on the extent to which sleep after learning benefits recognition memory tasks, it is important to quantify and compare the size of the sleep benefit across recognition and recall tasks. Thus, we entered memory type as a moderator in our meta-analysis.

Sleep has previously been found to improve emotional episodic memories more than neutral memories, with some studies suggesting a specific role of REM sleep in the consolidation of emotional memories (e.g., Groch et al., 2015; Payne & Kensinger, 2010; Wagner et al., 2001; see Kim & Payne, 2020 for a review). Two recent meta-analyses investigated the preferential role of sleep in emotional memory consolidation. Schäfer et al. (2020) found that sleep improved both emotional and neutral memory equally, with no evidence for preferential impact on emotional memory; in fact, the difference between emotional and neutral memory was larger after wake than sleep. However, Schäfer et al. (2020) did find that when their analysis was restricted to experiments that contrasted SWS and REM sleep, sleep that consisted primarily of REM did show a preferential consolidation effect for emotional memory, although the number of studies included in this analysis was small. Lipinska et al. (2019) also found no meta-analytic evidence in favor of sleep’s preferential impact on emotional memory. However, they did find that the preferential effect was larger in recall tasks compared to recognition tasks and in studies that controlled for initial learning. In the current meta-analysis, we shed new light on these issues by focussing on sleep deprivation manipulations. To investigate whether the effect of sleep deprivation on memory performance is modulated by emotionality of the to-be-remembered stimuli, we entered emotionality as a moderator in our meta-analysis.

## Impact of Total Sleep Deprivation Before Learning and Potential Moderators of the Effect

Recent research has also proposed a role for sleep *before* learning. According to the synaptic homeostasis hypothesis (Cirelli & Tononi, in press; Tononi & Cirelli, 2003, 2012), learning occurs during wake when a neuron detects a statistical regularity in its input and begins to fire in response to this regular input. In other words, successful learning requires neurons to be able to fire selectively in response to statistically regular patterns observed in the environment. To do so, strength of the synapses carrying these inputs must be increased. However, the neuron now faces the plasticity-selectivity dilemma. As an increasing number of input lines become strengthened, a larger range of input patterns can make the neuron fire, reducing the neuron’s ability to fire selectively. This loss of selectivity corresponds to reduced ability to encode new information. During sleep, the brain spontaneously activates both new information encoded during previous wake and information encoded in the past. Over the course of this activation, those synapses that are activated most strongly and consistently during wake survive, while at the same time, those synapses that were less activated are weakened. This weakening occurs primarily during the transitions between intracellular up and down states experienced during SWS. This competitive down-selection of weaker synapses restores memory encoding ability.

The restorative function of sleep is supported by evidence showing decreased episodic learning ability across a 6-hr retention interval in which participants remained awake, whereas encoding capacity was restored after a daytime nap (Mander et al., 2011). Further, neuroimaging evidence suggests sleep deprivation prior to learning is associated with disrupted encoding-related functional activity in the bilateral posterior hippocampus (Yoo et al., 2007; for similar findings, see Alberca-Reina et al., 2015; Drummond & Brown, 2001; Van Der Werf et al., 2009). Thus, sleep deprivation before learning may be detrimental specifically for the encoding of hippocampal-dependent declarative memories. Our second meta-analysis seeks to estimate the effect size associated with this impairment. As only two studies have looked at the impact of sleep deprivation on procedural learning when it occurs after deprivation, we were not able to assess the potential moderating effect of declarative versus procedural memory. We were also not able to use emotionality as a moderator here due to the low number of relevant studies. Yet, there are several studies that have used recall tasks and recognition memory tasks, so we were able to evaluate the moderating effects of recall versus recognition, as in the first meta-analysis.

## The Present Meta-Analyses

Despite the breadth of evidence for an effect of total sleep deprivation both before and after learning on memory performance, there is no comprehensive review and analysis of the strength of the effect of sleep deprivation on long-term memory. Previous reviews and meta-analyses investigating a role of sleep deprivation have focused on tasks that are likely more susceptible to fatigue. Pilcher and Huffcutt (1996) conducted a meta-analytic review of the effect of sleep deprivation on cognitive and motor task performance in 19 primary studies and found that sleep deprivation had a significant impact on performance. Still, this meta-analysis does not address long-term memory performance. Similarly, Lim and Dinges (2010) found an effect of sleep deprivation on a range of cognitive tasks including attention, working memory, and short-term memory, though the size of the effect varied depending on the task (e.g., a nonsignificant effect on reasoning accuracy, but a large effect on attention). Finally, Harrison and Horne (2000b) in a review found that sleep deprivation impacted decision-making ability. The tasks studied in these reviews are often repetitive and monotonous (e.g., the Psychomotor Vigilance Task; Dinges & Powell, 1985, the go/no go paradigm, and tests of serial addition), and they tend to probe lower-level functions that are particularly susceptible to fatigue, such as reaction times and processing speed. Therefore, the conditions of these studies are arguably better suited to finding adverse effects of sleep deprivation on performance than studies looking at higher-level learning and long-term memory. Thus, a review of the effects of sleep deprivation on such high-level, long-term memory is required.

Taking a meta-analytic approach will permit not only a quantitative assessment of the size of the main effect of sleep deprivation and its moderators but also an investigation of methodological quality within this literature including the statistical power of studies proposing to find a sleep deprivation effect. Variety in sample selection and methodological designs used within this literature raises the possibility of variations in methodological quality. Such variations could lead to biases in the meta-analysis by overestimating or underestimating the effect size (Higgins et al., 2011). Thus, we developed a checklist to assess multiple aspects of methodological quality, including questions specifically relevant to the assessment of sleep effects (e.g., excluding participants with sleep disorders), questions on study design (e.g., within-subject vs. between-subjects design, and random allocation to conditions), and questions on data analysis (e.g., preregistration and a priori power analyses). We used similar questions to other meta-analyses in the sleep literature (Lim & Dinges, 2010; Lo, Groeger, et al., 2016; Schäfer et al., 2020) and examined methodological quality as a continuous moderator in the analysis. The full methodological quality checklist is provided in Supplemental Appendix A.

It has been suggested that psychological science more broadly is currently suffering from a replication crisis due to low power, publication bias, selection biases, and analysis errors (Nosek et al., 2015). Low power limits potential to detect genuine effects but also results in Type I errors and exaggerated effect sizes (Ioannidis, 2005; Pollard & Richardson, 1987). Szucs and Ioannidis (2017) conducted an analysis of almost 4,000 cognitive neuroscience and psychology papers and found that the overall mean power to detect small, medium, and large effects was 17%, 49%, and 71%, respectively, with even lower power in the subfield of cognitive neuroscience. Given the convention that power to detect an effect size should be at least 80% (Di Stefano, 2003), it is clear that a large number of studies within psychology are underpowered (see Button et al., 2013; for further evidence of low statistical power within neuroscience). In the sleep literature, sample sizes tend to be low, potentially due to the resource intensity of conducting these experiments. Thus, we investigated whether the low power seen more broadly in psychological science and neuroscience is also evident in the sleep deprivation literature. For each individual effect size entered into the meta-analysis, we calculated the study’s power (defined as power to detect our meta-analytic effect size) and investigated whether there is an association between a study’s power and the effect size observed in the study.

## Method

### Search Strategy

For study selection, we generated the Boolean search term “Sleep AND (deprivation OR restriction OR loss) AND (learning OR memory OR conditioning)” and conducted a search in the electronic databases EBSCOhost (included PsycARTICLES, PsycEXTRA, PsycINFO, and PsycTESTS) and PubMED on July 29th, 2020. This search yielded 2,213 empirical articles published between January 01, 1970 and July 29, 2020 in peer-reviewed journals in English using human participants.

In line with best practice guidelines (Rothstein et al., 2005; Siddaway et al., 2019), we ran several searches on July 13th, 2020, using the same search terms as above, to identify gray literature in an attempt to mitigate against publication bias. These searches yielded a total of 553 items. Specifically, we widened our search criteria in EBSCOhost and PubMED to include unpublished dissertations and theses, conference materials, and preprints; we searched the bioRxiv and PsyArXiv repositories for preprints; and we searched the ProQuest and OpenGrey (a European database in which national libraries submit unpublished studies) databases for unpublished dissertations and theses, conference materials, and for research grants and fellowship awards. Additionally, we contacted all authors who had published data included in our initial screening results to ask for unpublished data that fit our inclusion criteria, and this yielded one preprint article. We also had one in-press article during the search period that fits our inclusion criteria (Tamminen et al., 2020) and was therefore included in our search results. In sum, we identified both published and unpublished data with search strategies spanning (a) peer-reviewed published articles, (b) in-press articles, (c) preprints uploaded to repositories, (d) unpublished dissertations and theses, (e) conference materials, and (f) research grants and fellowship awards.

**Figure 1 fig1:**
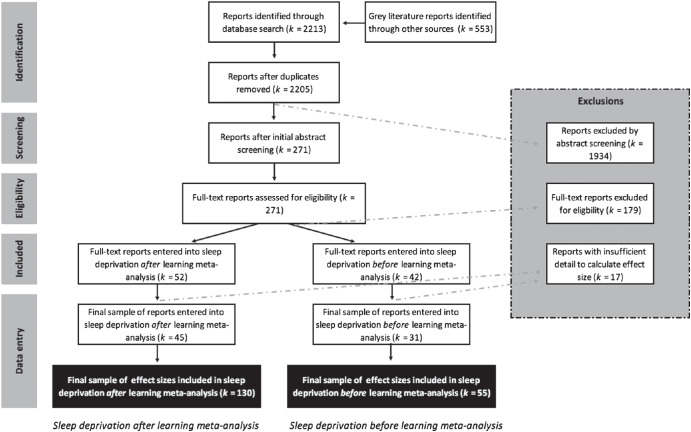
Flowchart Displaying Literature Search Process for the Deprivation After Learning and Deprivation Before Learning Meta-Analyses

We scanned the abstracts and full texts of all articles according to our inclusion and exclusion criteria, and separated them into articles that investigated the effect of sleep deprivation *after* learning, and those that investigated sleep deprivation *before* learning. Figure 1 displays a screening process flowchart showing that after exclusions were removed, 130 effect sizes (extracted from 45 reports) were included in the sleep deprivation after learning meta-analysis and 55 effect sizes (extracted from 31 reports) were included in the sleep deprivation before learning meta-analysis. The number of effect sizes included in each meta-analysis is greater than the number of full-text articles that fit our inclusion criteria. The reason for this is that several studies measured performance differences between a sleep deprivation and sleep control group using multiple tasks, multiple conditions (e.g., stimulus valence or procedural instructions), and across multiple time points. Effect sizes were calculated for each of these data sets within an article because each variation represents a different, yet correlated, measurement of the impact of sleep deprivation on memory. However, there were some studies that used multiple outcome measures to assess performance in a single task (e.g., accuracy and reaction time). Given that multiple outcome measures within the same task are different ways of assessing the same manipulation, we chose only one outcome measure for calculating an effect size in these instances, according to the following hierarchy from most to least preferred outcome measure: accuracy as measured by retention performance (i.e., performance change from training to test), accuracy at test only, reaction time measured by retention performance (i.e., performance change from training to test), and reaction time at test only. Further, in recognition tasks, if both signal detection analyses and analyses based on proportion correct were reported, we chose to include the signal detection measure only. For example, if a study reported both *d*-prime and reaction times in a recognition memory task (e.g., Tamminen et al., 2020), we only used the *d*-prime data.[Fig fig1]

A list of studies included in the two meta-analyses and their key properties can be found in Supplemental Appendix B (studies investigating sleep deprivation after learning) and Supplemental Appendix C (studies investigating sleep deprivation before learning), as well as at osf.io/5gjvs/.

### Inclusion/Exclusion Criteria

To select relevant studies, we applied the following inclusion/exclusion criteria.

a Participants had to be healthy adults aged 18 years and older.
b Studies must have included, as a primary independent variable, a manipulation of sleep deprivation that was a minimum of one night of total sleep deprivation with an appropriate sleep control condition consisting of one normal night of sleep. Residency studies (studies conducted in a medical setting) were excluded due to the lack of control over whether total sleep deprivation occurred (sleep deprivation is often reported despite naps having occurred on shift; e.g., Bartel et al., 2004; Guyette et al., 2013). Additionally, studies using sleep restriction protocols, which involve multiple nights of limited sleep duration rather than one or more nights of no sleep, were excluded because the neural and cognitive effects of sleep restriction may differ from those caused by total sleep deprivation (Banks & Dinges, 2007; Lowe et al., 2017).
c Studies must have included, as a primary dependent variable, at least one measure of learning or long-term memory where the task was described in sufficient detail to ascertain which cognitive function it assessed.
d For the meta-analysis investigating sleep deprivation after learning, the cognitive task must have had a single encoding phase and a retrieval phase(s) that were temporally separated by either a period of sleep deprivation or an equivalent period of sleep. For the meta-analysis investigating sleep deprivation before learning, the single encoding phase and the retrieval phase(s) must have been temporally separated by a retention interval that had a minimum duration of at least 1 min, rather than being part of the same task session. The reason for this criterion is that our meta-analyses aimed to investigate effects of sleep deprivation on learning and long-term memory. The inclusion of studies with temporally indistinct encoding and retrieval phases would have included short-term and working memory tasks that form a separate body of literature (Lim & Dinges, 2010), the analysis of which was beyond the scope of these meta-analyses.
e In cases in which studies assessed the effects of other interventions (e.g., caffeine; Kilpeläinen et al., 2010) in ameliorating sleep deprivation effects, studies were included only if data could be obtained from the control sleep deprivation and control sleep groups. This criterion was included because the goal of the current meta-analyses was to assess effects of sleep deprivation in the absence of alertness-promoting strategies.
f Studies must have reported sufficient statistical detail to calculate effect sizes (means, *SD*, *F*, and *t*). When statistical details were not reported in the text, we either contacted corresponding authors to request relevant data or we extracted the data needed from published figures in the article using WebPlotDigitizer (Rohatgi, 2019).

### Methodological Quality

Through our survey of the literature, it became clear that sleep deprivation studies differ considerably in various aspects of methodological rigor (e.g., lack of control over adherence to sleep manipulations in the sleep deprivation and sleep control groups; Fischer et al., 2002 vs. complete control; Chatburn et al., 2017). For this reason, we assessed the methodological quality of each study entered into our meta-analyses and included this in our moderator analyses.

To assess methodological quality, we developed a 22-item checklist based on criteria for standard sleep deprivation experiments (e.g., preexperimental sleep monitored using actigraphy and exclusion of sleep disorders) and more general experimental psychology experiments (e.g., a priori power analysis and study design). For each item on the checklist, studies were scored with either a zero or a one according to whether they satisfied the criterion. To transform the total methodological quality score for each study into a risk of bias that reflects a rank of all the studies on a common scale, we normalized the total scores by dividing each study’s total methodological quality score by the maximum total methodological quality score that was achieved among all studies (Stone et al., 2020). Lower values imply lower ranked studies (minimum score of 0) and higher values imply higher ranked studies (maximum score of 1) relative to the best study. The full methodological quality checklist can be found in Supplemental Appendix A.

Given that the checklist items form a multidimensional scale, the items were clustered according to the Downs and Black’s (1998) instrument for assessing methodological quality, which assesses five types of bias: “reporting,” “internal validity—bias,” “internal validity—confounding,” “power,” and “external validity.” The “reporting” cluster determines whether sufficient information is provided to make an unbiased assessment of study findings. In our methodological quality checklist, the items in this cluster referred to the reporting of exclusion criteria for participant characteristics (e.g., “Did the study exclude participants with a history of sleep disorders?”). The “internal validity—bias” cluster assesses whether biases were present in the intervention or outcome measure that would favor one experimental group [e.g., “Was interference for the sleep deprivation group low (nondemanding activities given and monitored in the lab)?”]. The “internal validity—confounding” cluster assesses whether biases were present in the selection and allocation of participants (e.g., “For within-group studies, was the order of deprivation and control conditions counterbalanced?”). The “power” cluster assesses whether a study used a priori power analyses to avoid Type II errors (e.g., “Did the study report an a priori power analysis with power set at 80% or higher and an α at .05 or lower?”). The Downs and Black’s (1998) “external validity” cluster determines the extent to which findings can be generalized to the population from which a sample was taken (e.g., “Were the staff, places, and facilities where the patients were treated, representative of the treatment the majority of patients receive?”; Downs & Black, 1998, p. 383). Since the items in this cluster were designed for clinical intervention studies with nontypical populations, we dropped the external validity cluster from our checklist. In line with Cochrane Collaboration recommendations (Higgins et al., 2011), the four clusters in our methodological quality checklist (hereon referred to as reporting, bias, confounding, and power) were then included in moderator analyses. The percentage of studies that passed on each item of the quality checklist for both Meta-Analysis 1 and Meta-Analysis 2 can be found in Supplemental Appendix D. Total methodological quality scores for each study, as well as the item-level ratings, can be found at osf.io/5gjvs/.

### Effect Size Calculation

Information on study means, standard deviation, and effect sizes for each item, as well as formulas used to calculate effect sizes, can be found at osf.io/5gjvs/. We report the standardized mean difference in task performance between a sleep deprivation and sleep control group, with positive values indicating that sleep deprivation influenced learning and memory such that performance was significantly worsened compared to a sleep control group. For studies with independent samples (between-subjects designs), we computed Cohen’s *d*_*s*_ based on the means and variance reported in each study for the sleep and sleep deprivation group. For within-subject designs, in which participants took part in both the sleep deprivation and sleep control conditions, we calculated Cohen’s *d*_*av*_, as recommended by Lakens (2013).

### Data Analysis

#### Overall Meta-Analytic Effect Size

All analysis code can be found at osf.io/5gjvs/. To analyze whether there was an overall meta-analytic effect of sleep deprivation versus overnight sleep on memory performance, we fitted a multilevel random-effects model using the R package metafor (Viechtbauer, 2010). A random-effects model allows for inconsistencies between effect sizes from varying study designs, assuming systematic variability between effect sizes in addition to random sampling error. A random-effects model therefore provides more conservative effect size estimates than a fixed-effect model (Borenstein et al., 2010). A multilevel model allows for the inclusion of both within-study effect sizes and between-study effect sizes (Assink & Wibbelink, 2016). Many experiments included in the meta-analysis report multiple dependent effect sizes, such as results from multiple test sessions, multiple within-group experimental conditions (e.g., performance on emotional vs. neutral stimuli), or multiple outcomes (e.g., a procedural and declarative memory task). Including multiple dependent effect sizes from the same experiment violates the assumption of data independence assumed in a typical random-effects model. A multilevel meta-analysis accounts for such dependencies by modeling both within-study and between-study effects. Thus, we were able to model variance accounted for by (a) random error, (b) within-study differences among effect sizes within the same experiment, and (c) between-study differences across different experiments.

#### Heterogeneity

To investigate whether moderating variables may influence the size of the effect of sleep deprivation, we examined heterogeneity within the data set using the *Q* test (Cochran, 1954). The *Q* test indicates whether there is heterogeneity within the data set and is calculated by the weighted sum of the squared deviations of individual study effect estimates and the overall effect across studies. Significant heterogeneity suggests that some of the variance within the data set may not be due to random sampling error, and thus moderating variables may influence the effect. Since we were interested in both the within-study and between-study variance, we ran two separate one-sided log-likelihood-ratio tests. As such, the fit of the overall multilevel model was compared to the fit of a model with only within-study variance and to a model with only between-study variance. This allowed us to determine whether it was necessary to account for both within- and between-study variances within our model.

Assink and Wibbelink (2016) suggest that such log-likelihood ratio tests may be subject to the issues of statistical power when the data set comprises a small number of effect sizes. Low statistical power may lead to nonsignificant effects of heterogeneity when in fact there is variance within or between studies. To account for this, it is recommended to also calculate the *I* ^2^ statistic, which indicates the percentage of variation across studies that is due to heterogeneity and that which is due to random sampling error (Higgins & Thompson, 2002). Hunter and Schmidt (2004) suggest the 75% rule, such that if less than 75% of overall variance is attributed to sampling error, then moderating variables on the overall effect size should still be examined. Using the formula of Cheung (2014), we calculated the percentage of variance that can be attributed to each level of our model.

However, although *I* ^2^ reports the proportion of variation in observed effect sizes, it does not provide us with absolute values that tell us the variance in true effects (Borenstein et al., 2017). Thus, as recommended by Borenstein et al. (2011), we report the τ^2^, which provide an estimate of the true effect size, and we report prediction intervals, which indicate that 95% of the time, effect sizes will fall within the range of those prediction intervals.

#### Publication Bias

To assess publication bias, we first examined a contour enhanced funnel plot. Funnel plots show each effect size plotted against its standard error, with contour lines corresponding to different levels of statistical significance. If studies are missing almost exclusively from the white area of nonsignificance, then there may be publication bias. If studies are missing from areas of statistical significance, the bias is likely due to other causes such as poor methodological quality, true heterogeneity, chance, or the bias may be artifactual (Johnson, 2021; Sterne et al., 2011). We also conducted a variation of Egger’s regression test for funnel plot asymmetry (Egger et al., 1997) that can be conducted with multilevel meta-analyses.

## Results

### Meta-Analysis 1: Sleep Deprivation After Learning

This meta-analysis summarizes research from 45 reports investigating effects of sleep deprivation after learning (130 effect sizes) published in English between 1994 and 2020 across a total of 1,616 participants. All reports used healthy adult populations and deprived participants of one night of sleep postlearning. Notably, two reports from this meta-analysis also report data that are relevant to the sleep deprivation before learning meta-analysis (Fischer et al., 2002; Tamminen et al., 2020). See Table 1 for central tendencies and frequency data for moderator and descriptive variables of studies included. The table shows that this literature is heavily biased toward young adults, severely limiting conclusions that can be drawn about older age groups. The literature predominantly uses between-groups designs rather than the statistically more powerful within-group designs, partly explaining and exacerbating the low power highlighted later in our analysis. Recognition memory and recall memory tasks are the most often employed measures of memory, and most of the literature probes declarative memory rather than procedural memory. We return to these memory type distinctions in our moderator analyses. Most studies used human observation to ensure participants in the sleep deprivation condition did not sleep during the night, but few ensured that they did not sleep during the day to the same standard. Low compliance during the day could possibly dilute the effect size. Finally, most but not all studies allowed recovery sleep after deprivation. We return to this in the moderator analyses.[Table tbl1]

#### Overall Meta-Analytic Effect Size

**Figure 2 fig2:**
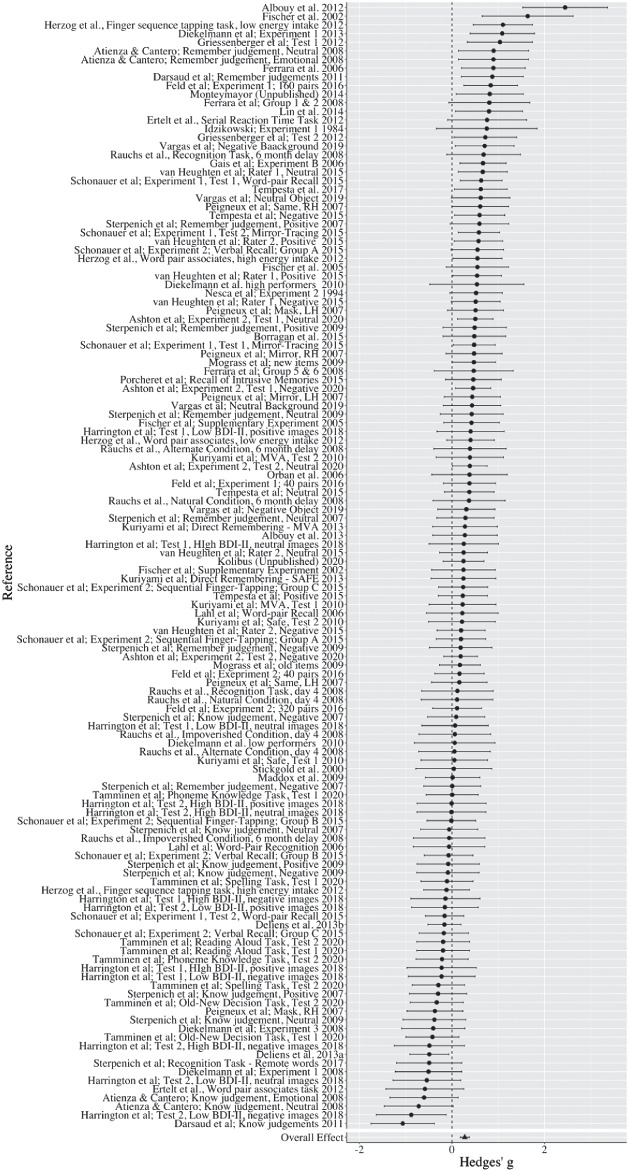
Forest Plot Containing Effect Sizes and 95% Confidence Intervals for the Difference in Performance Between a Sleep Deprivation and Sleep Control Group on Memory *Note*. Effect sizes to the right indicate an effect of sleep deprivation after learning such that memory was significantly worse than in a sleep control group.

The overall effect size for the mean difference in memory performance between the sleep deprivation and sleep control group, measured by Hedges’ *g*, was 0.277 (*SE* = 0.050), indicating a small-to-medium effect according to Cohen’s categorization, and a significant difference from zero, 95% CI [0.177, 0.377], *p* < .001. Figure 2 displays a forest plot of the effect sizes. See Supplemental Appendix B for a summary of all studies included in the meta-analysis.[Fig fig2]

Some of the variance within the data set could not be explained by random error, highlighted by overall significant heterogeneity, *Q*(129) = 244.891, *p* < .001. An analysis of heterogeneity of between-study variance (Level 2) revealed a significant difference between a full and a reduced model (*p* < .001), suggesting significant variability between studies. An analysis of heterogeneity of within-study variance (Level 3) also revealed a significant difference between a full model and a reduced model (*p* < .001), suggesting significant variability between within-study effect sizes. We further calculated the *I* ^2^ statistic, which indicates the percentage of variance that could be attributed to each level of the model. Using the formula from Cheung (2014), approximately 52% of variance can be attributed to sampling error, 14% to within-study variance, and 34% to between-study variance. Next, we calculated τ^2^, which provides a measure of the variance of the true effects; τ^2^ = .026 for within-study variance, and τ^2^ = .061 for between-study variance. Prediction intervals indicated that 95% of effect sizes would fall in the range of −0.316 and 0.870.

Based on the significant heterogeneity between studies, the large prediction intervals, as well as the 75% rule, such that moderators should be explored if less than 75% of the variance can be attributed to random sampling error (Hunter & Schmidt, 2004), we therefore explored the effect of potential moderating variables on the direction of the effect.

#### Publication Bias

**Figure 3 fig3:**
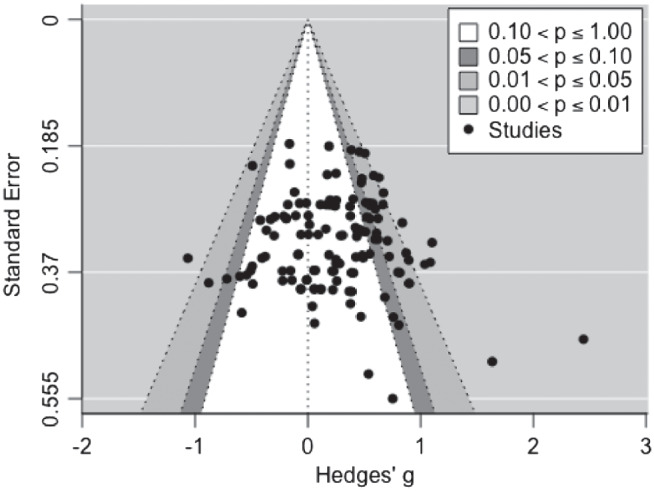
Contour Enhanced Funnel Plot Showing the Hedges’ g Effect Size on the x-Axis, and the Standard Error of Hedges’ g Effect Size on the y-Axis

Figure 3 shows a funnel plot of the effect sizes. Visual inspection of the funnel plot indicates that effect sizes are not evenly distributed across the funnel plot, raising the potential for publication bias in which studies reporting a positive effect are more likely to be published. Egger’s regression test reveals significant funnel plot asymmetry (*z* = 2.297, *p* = .022), supporting this assessment of the funnel plot. Further visual inspection of the funnel plot reveals two potential outlier effect sizes in the area of high statistical significance; these potential outliers are characterized by large effect sizes and large standard error (therefore smaller sample sizes). These large effect sizes on the right-hand side of the plot suggest that there may be a bias in this literature toward publishing significant effects, regardless of the precision with which the study effect size can be estimated. However, the presence of multiple studies in the area of nonsignificance suggests other biases may also contribute to the asymmetry.[Fig fig3]

Because Egger’s test indicates the presence of publication bias, we sought to quantify the impact of this bias on the estimated effect size. We conducted a trim-and-fill analysis (Duval & Tweedie, 2000), which calculates potential missing effect sizes to create a symmetric funnel plot and then provides an adjusted overall meta-analytic effect size based on this funnel plot symmetry. Although this is a well-used method to assess publication bias, it assumes that effect sizes are independent of each other. The current meta-analysis uses a multilevel approach, with dependencies between some effect sizes. The adjusted effect size should therefore be considered a preliminary estimate. The trim-and-fill method estimated 12 missing studies from the left-hand side of the funnel plot. With these effect sizes included, the adjusted overall meta-analytic effect size was smaller than the original effect size of *g* = 0.277, although it was still significantly greater than zero, Hedges’ *g* = 0.166, *SE* = 0.041, 95% CI [0.086, 0.247], *p* < .001.

#### Outlier Analysis

We explored whether outliers and influential cases may have significantly influenced the meta-analytic effect size. To identify the presence of outliers, we identified any effect sizes with studentized residuals greater than or smaller than three, which identified one effect size as an outlier (Albouy, Sterpenich, et al., 2013). However, an outlier may not necessarily influence the size of the overall effect (Viechtbauer & Cheung, 2010). Therefore, based on suggestions by Viechtbauer and Cheung, we conducted influential case analyses, to identify any effect sizes that exerted a significant influence on the size of the overall meta-analytic effect. We measured Cook’s distance to examine the influence of deleting each study on the overall size of the effect, and DFBETAs to examine the effect of deleting each study on individual parameter estimates. Cook’s analysis identified a further one effect size that was found to be an influential case (Darsaud et al., 2011, Know judgements). Removal of the outlier and influential case reduced the overall meta-analytic effect size to 0.271 (from 0.277). Since moderator analyses examine smaller subsets of effect sizes, we removed these two specific effect sizes from all moderator analyses conducted.

#### Moderator Analysis

We introduced four categorical moderating variables and analyzed the effect of each moderator separately on the size of the effect of sleep deprivation on learning and memory: (a) whether it was a declarative (*k* = 108) or procedural (*k* = 20) memory task, (b) for declarative tasks, whether task type was recall (*k* = 42) or recognition (*k* = 59), (c) whether participants received one or more recovery nights of sleep (*k* = 83) or no recovery sleep (*k* = 45), and (d) for those studies that investigated emotionality, whether the stimuli were emotional (*k* = 31) or neutral (*k* = 20). Supplemental Appendix B shows the classification of each study on these dimensions.

Whether participants received a recovery night of sleep before the test session as a moderator had a significant effect, *Q*(1) = 10.496, *p* < .001. Thus, we ran separate effect size analyses for those studies where participants had a night of recovery sleep and those that did not. For those studies that had one or more nights of recovery sleep, there was a small effect of sleep deprivation on learning and memory, Hedges’ *g* = 0.176 (*SE* = 0.058), which is significantly different from zero, 95% CI [0.060, 0.292], *p* = .003; *Q*(82) = 133.766, *p* < .001. For those studies that did not have a night of recovery sleep, the effect size was larger, Hedges’ *g* = 0.410, *SE* = 0.044, 95% CI [0.320, 0.499], *p* < .001; *Q*(44) = 50.042, *p* = .246.

Whether the task probed declarative or procedural memory also had a significant moderating effect, *Q*(1) = 5.301, *p* = .021. We therefore ran separate effect size analyses for those studies that implemented a declarative memory task and those that implemented a procedural memory task. For those studies with a declarative memory task, there was a small effect of sleep deprivation on learning and memory, Hedges’ *g* = 0.218 (*SE* = 0.055), which is significantly different from zero, 95% CI [0.109, 0.327], *p* < .001; *Q*(107) = 174.802, *p* < .001. For those studies with a procedural memory task, the effect size was larger, Hedges’ *g* = 0.449, *SE* = 0.083, 95% CI [0.276, 0.623], *p* < .001; *Q*(19) = 24.794, *p* = .167.

Since we found a significant moderating effect of both recovery sleep and task type (declarative or procedural), we ran a further analysis to investigate whether there was an interaction between the two significant moderators. The analysis revealed no significant interaction between recovery sleep and memory type, *Q*(1) = 0.804, *p* = .370, suggesting that whether participants received recovery sleep or not affected declarative and procedural memory task performance in a similar way. Whether studies used a recall or recognition task did not have a significant effect on the size of the effect of sleep deprivation on learning and memory. Studies using a recall task had a mean effect size of Hedges’ *g* = 0.209, *SE* = 0.082, 95% CI [0.045, 0.374], *p* = .014; *Q*(41) = 76.317, *p* < .001, and studies with a recognition task had an overall effect size of Hedges’ *g* = 0.175, *SE* = 0.077, 95% CI [0.021, 0.330], *p* = .027; *Q*(58) = 91.950, *p* = .003. Effect sizes associated with emotional and neutral stimuli were also not significantly different, with an overall effect size of Hedges’ *g* = 0.251, *SE* = 0.080, 95% CI [0.084, 0.417], *p* = .005; *Q*(19) = 25.789, *p* = .136, for studies using neutral stimuli, and an overall effect size of Hedges’ *g* = 0.207, *SE* = 0.085, 95% CI [0.033, 0.380], *p* = .021; *Q*(30) = 41.888, *p* = .073, for emotional stimuli.

**Figure 4 fig4:**
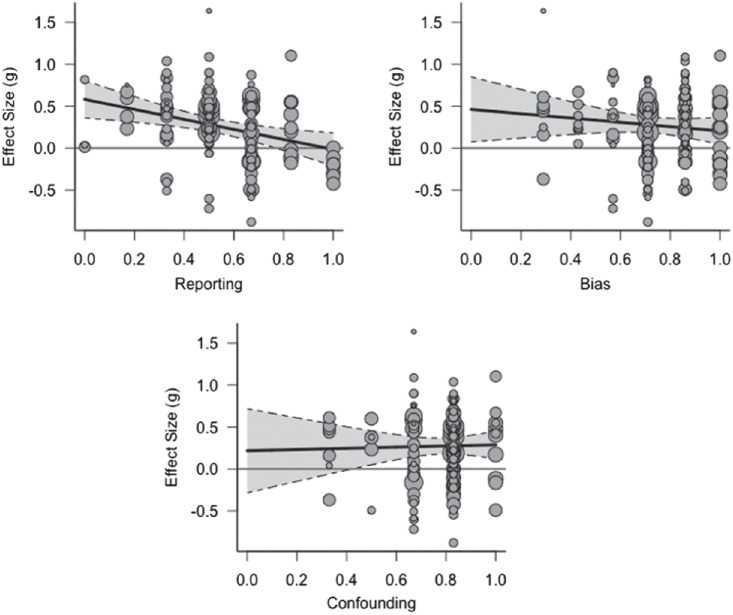
Meta-Analytic Scatter Plot With Methodological Quality of the Reporting, Bias, and Confounding Clusters Plotted Against Individual Study Effect Size for Meta-Analysis 1 *Note*. The size of each point is proportional to the weight the study received in the analysis, with larger size indicating larger weight. The solid regression lines represent the effect size predicted by the meta-regression model as a function of each cluster score, with corresponding 95% confidence intervals.

We then introduced four continuous moderating variables and analyzed the effect of each moderator separately on the size of the effect of sleep deprivation on learning and memory: (a) methodological quality reporting cluster, (b) methodological quality bias cluster, (c) methodological quality confounding cluster, and (d) statistical power to find the mean effect size established in the meta-analysis (*g* = 0.277). Since methodological quality was divided into clusters in our methodological quality checklist, we introduced these clusters (reporting, bias, and confounding) as moderators. We did not include the power cluster as a moderator, since the majority of studies did not calculate power and thus scored zero on this cluster, with only one study (contributing eight effect sizes) providing a power analysis. For each of the methodological quality clusters, we created a meta-analytic scatter plot using the metafor package (Viechtbauer, 2010), showing the Hedges’ *g* of each individual study plotted against each moderator (see Figure 4). The figure shows that an effect size of zero falls within the 95% confidence interval in the reporting cluster for studies scoring 0.8 or higher, suggesting that these studies show no effect of sleep deprivation while the lower scoring studies do. Consistent with this observation, the reporting cluster was a significant moderator, *Q*(1) = 9.214, *p* = .002. Visual inspection of the plot for the confounding cluster suggests that studies with scores of 0.3 or lower on this cluster may not show an effect of sleep deprivation. However, this cluster did not show a statistically significant moderating effect, perhaps because there were no studies that scored below 0.3 on this cluster. Bias did not show a significant moderating effect either.[Fig fig4]

**Figure 5 fig5:**
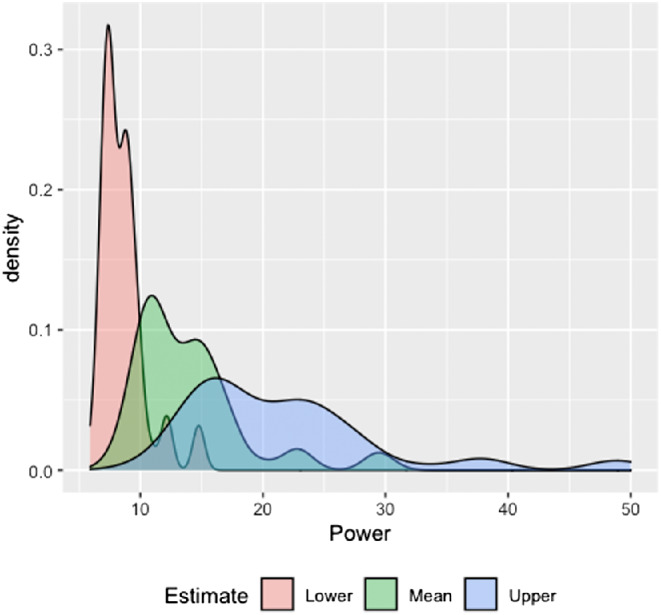
Distribution of Power to Find the Mean Meta-Analytic Effect Size (Green), the Lower Bound of the 95% Confidence Interval Around the Mean (Pink), and the Upper Bound of the 95% Confidence Interval Around the Mean (Blue) *Note*. See the online article for the color version of this figure.

To assess the achieved statistical power of each individual experiment to detect the mean meta-analytic effect size, we conducted a post hoc power analysis in G*Power (Faul et al., 2007). For each study, we calculated the power to detect the mean meta-analytic effect size (*g* = 0.277), as well as the power to detect the 95% upper and lower confidence intervals of the effect size. The distribution of the mean power and lower and upper confidence interval bounds of the power estimate are plotted in Figure 5. We found the mean power to find the average meta-analytic effect to be 13.98% (*SD* = 4.60%, range = 7.2%–30.2%). The moderator analysis revealed no significant effect of power on the size of the meta-analytic effect. All moderator analyses are reported in Table 2.[Fig fig5] [Table tbl2]

### Meta-Analysis 2: Sleep Deprivation Before Learning

This meta-analysis summarizes research from 31 reports investigating effects of sleep deprivation before learning (55 effect sizes) published in English between 1989 and 2020 across a total of 927 participants. All reports used healthy adult populations and deprived participants of one night of sleep prior to learning. Notably, two reports from this meta-analysis also include data that are relevant to the sleep deprivation after learning meta-analysis (Fischer et al., 2002; Tamminen et al., 2020). See Table 3 for central tendencies and frequency data for moderator and descriptive variables of reports included. Again, the literature mostly involves young adults, leaving a gap in our understanding of how the effects of interest change with age. The discrepancy between use of between- and within-group designs is lower here than in the first meta-analysis, and recognition memory and recall memory tasks are roughly equally represented. However, nearly all studies look at declarative memory, suggesting that more work on procedural memory is needed. While most studies ensured compliance with the sleep deprivation manipulation with direct observation at night, few did so during the preceding day, potentially diluting the impact of sleep deprivation.[Table tbl3]

#### Overall Sleep Deprivation Effect Size

**Figure 6 fig6:**
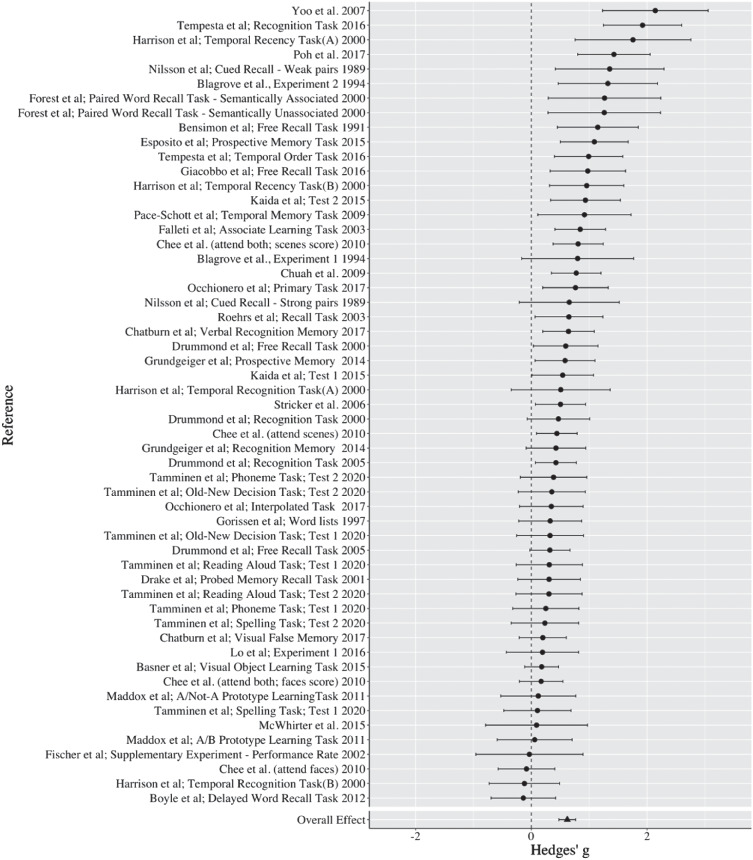
Forest Plot Containing Effect Sizes and 95% Confidence Intervals for the Difference in Performance Between a Sleep Deprivation and Sleep Control Group on Memory *Note*. Effect sizes to the right indicate an effect of sleep deprivation before learning such that memory was significantly worsened compared to a sleep control group.

The overall effect size for the mean difference in memory performance between the sleep deprivation and sleep control group, measured by Hedges’ *g*, was 0.621 (*SE* = 0.074), indicating medium to large effect according to Cohen’s categorization, and a significant difference from zero, 95% CI [0.473, 0.769], *p* < .001. Figure 6 provides a forest plot of the effect sizes. See Supplemental Appendix C for a summary of all studies included in the meta-analysis.[Fig fig6]

Some of the variance within the data set could not be explained by random error, highlighted by overall significant heterogeneity, *Q*(54) = 118.166, *p* < .001. An analysis of heterogeneity of between-study variance (Level 2) also revealed a significant difference between a full and a reduced model (*p* < .001), suggesting significant variability between studies. An analysis of heterogeneity of within-study variance (Level 3) revealed a significant difference between a full model and a reduced model (*p* < .001), suggesting significant variability between within-study effect sizes. The *I* ^2^ statistic indicates that approximately 41% of variance can be attributed to sampling error, 9% to within-study variance, and 50% to between-study variance. τ^2^ = .096 for between-study variance, and τ^2^ = .017 for within-study variance, and prediction intervals indicated that 95% of effect sizes would fall in the range of −0.069 and 1.312. Based on this evidence for heterogeneity, we explored the effect of potential moderating variables on the direction of the effect.

#### Publication Bias

**Figure 7 fig7:**
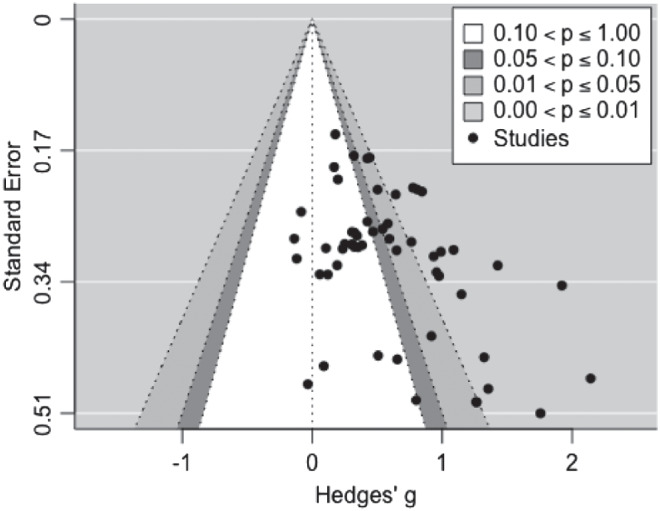
Contour Enhanced Funnel Plot Showing the Hedges’ g Effect Size on x-Axis, and Standard Error of Hedges’ g Effect Size on the y-Axis

Figure 7 shows a contour enhanced funnel plot of effect sizes. Similar to Meta-Analysis 1, visual inspection of the funnel plot indicates that effect sizes are not evenly distributed across the funnel plot, raising the potential for publication bias. Egger’s regression test supports this conclusion, indicating significant funnel plot asymmetry (*z* = 3.363, *p* < .001). Further inspection of the funnel plot indicates that the majority of effect sizes are clustered toward the right side of the funnel plot. While many of these studies fall in the area of nonsignificance, there appear to be studies missing from the left-hand side of the plot. It is possible these missing studies are due to researchers being unable to publish findings that contradict their hypotheses, and that the bias indicated by Egger’s test may therefore be due to publication bias rather than other types of bias.[Fig fig7]

Because Egger’s test indicates the presence of publication bias, we conducted a trim-and-fill analysis (Duval & Tweedie, 2000), in the same way as in the first meta-analysis. The trim-and-fill method estimated seven missing studies from the left-hand side of the funnel plot. With these effect sizes included, the adjusted overall meta-analytic effect size was smaller than the original effect size of *g* = 0.621, although it was still significantly greater than zero, Hedges’ *g* = 0.463, *SE* = 0.070, 95% CI [0.326, 0.601], *p* < .001.

#### Outlier Analysis

In the same way as the first meta-analysis, we explored whether any outliers and influential cases significantly influenced the size of the effect. We identified any effect sizes with studentized residuals greater than or smaller than three (*k* = 1; Tempesta et al., 2016, Recognition Task). Following recommendations by Viechtbauer and Cheung (2010), influential case analysis (Cook’s distance and DFBETAs) identified two further effect sizes that may have had a significant influence on the results (Poh & Chee, 2017; Yoo et al., 2007). Removal of the one outlier and two influential cases reduced the overall meta-analytic effect size to 0.525 (from 0.621). We removed these three specific outliers and influential cases from all moderator analyses.

#### Moderator Analysis

We introduced the categorical moderating variable task type, recall (*k* = 26) versus recognition (*k* = 20). We excluded studies that used a different task type, including a recency discrimination task (*k* = 3), a prototype learning task (*k* = 2), and a finger-tapping task (*k* = 1). Analysis of the moderator recall versus recognition revealed that the type of task used did not moderate the size of the effect, *Q*(1) = 0.028, *p* = .868. There were only two studies of procedural memory (Fischer et al., 2002; McWhirter et al., 2015) and only five entries where the participants were given a night of recovery sleep (two further entries did not report whether recovery sleep was given). Likewise, only two studies investigated the effects of sleep deprivation on emotional memory (Kaida et al., 2015; Tempesta et al., 2016). Thus, there was insufficient variability within the data set to assess whether one or more nights of recovery sleep, the type of memory (declarative, procedural), and emotionality moderated the size of the sleep deprivation effect.

**Figure 8 fig8:**
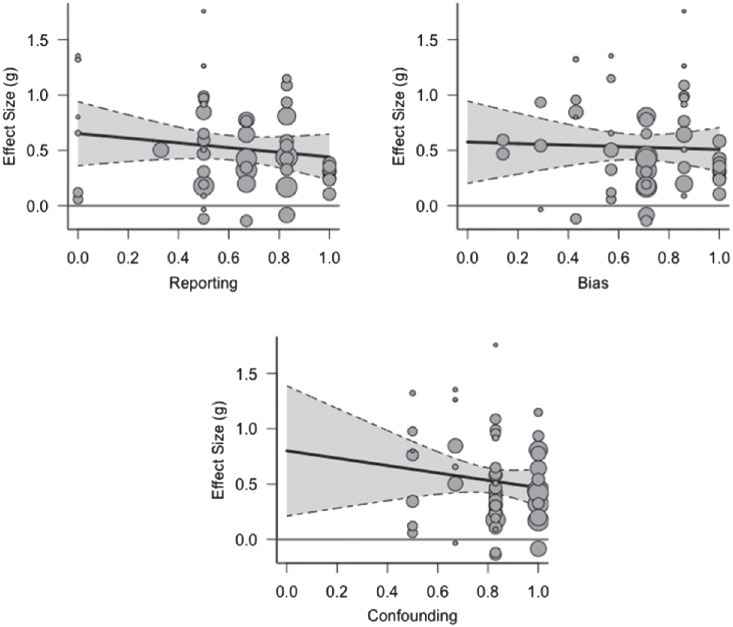
Meta-Analytic Scatter Plot With Methodological Quality Clusters of Reporting, Bias, and Confounding, Plotted Against Individual Study Effect Size for Meta-Analysis 2 *Note*. The size of each point is proportional to the weight the study received in the analysis, with larger size indicating larger weight. The solid regression lines represent the effect size predicted by the meta-regression model as a function of each cluster score, with corresponding 95% confidence intervals.

We tested the influence of the three continuous moderating variables assessing methodological quality (reporting, bias, and confounding), as well as statistical power to detect the meta-analytic effect size, on the size of the sleep deprivation effect. No methodological quality cluster had a significant moderating effect. For each of the methodological quality clusters, we created a meta-analytic scatter plot (metafor package; Viechtbauer, 2010; see Figure 8). We did not include the power cluster as a moderator, since the majority of studies did not calculate power and thus scored zero on this cluster, with only two studies (contributing a total of 10 effect sizes) providing a power analysis.[Fig fig8]

**Figure 9 fig9:**
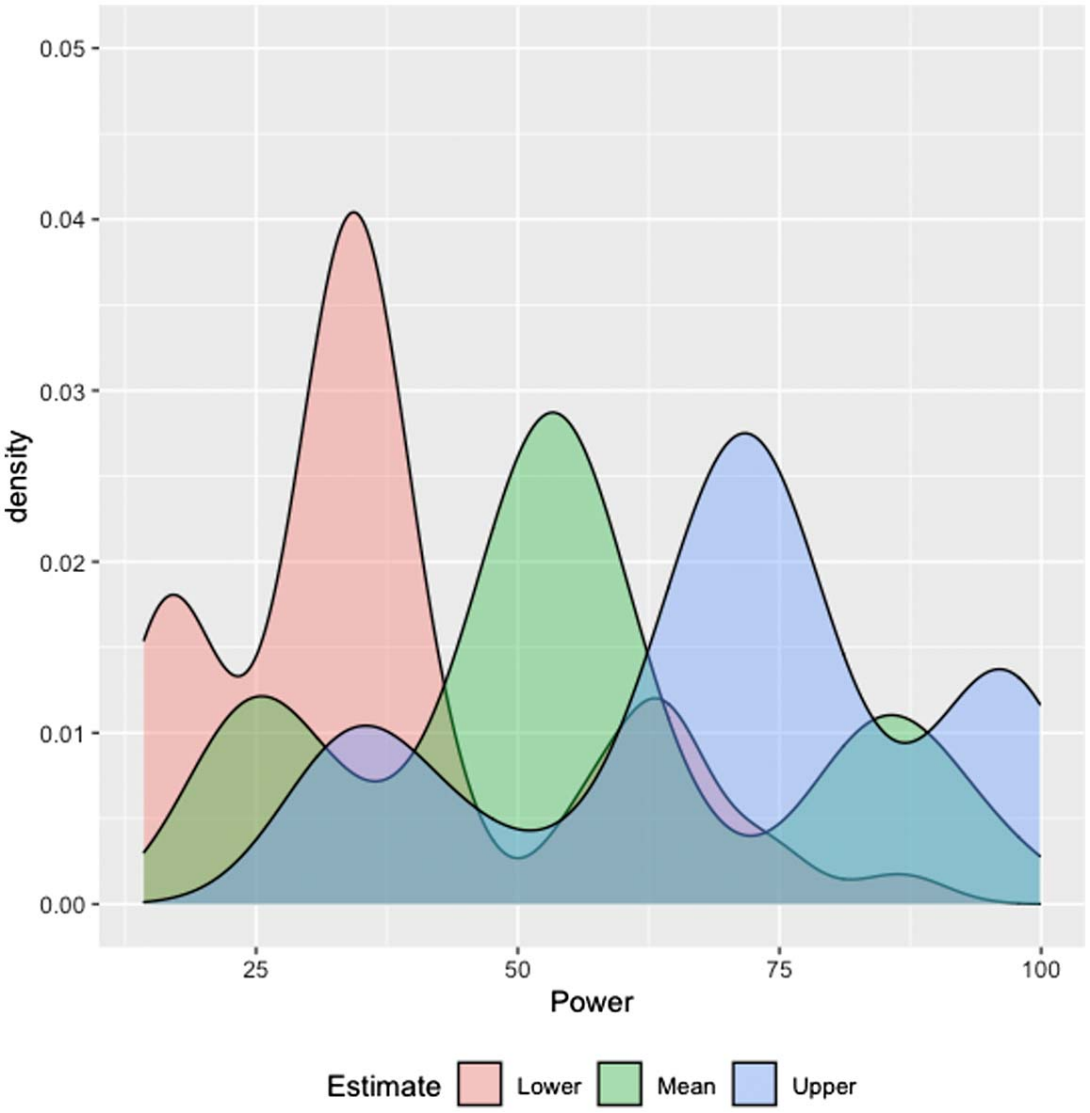
Distribution of Power to Find the Mean Meta-Analytic Effect Size (Green), the Lower Bound of the 95% Confidence Interval Around the Mean (Pink), and the Upper Bound of the 95% Confidence Interval Around the Mean (Blue) *Note*. See the online article for the color version of this figure.

We then investigated whether statistical power to find the meta-analytic effect moderated the size of the effect of sleep deprivation on memory. In the same way as in the first meta-analysis, for each study, we calculated the power to find the mean meta-analytic effect size, as well as the power to detect the upper and lower confidence interval bounds around the mean. The distribution of power to detect the three estimates is plotted in Figure 9. We found the mean power to find the meta-analytic effect to be larger than in the first meta-analysis (*M* = 54.77%, *SD* = 20.81%, range = 21.22%–98.06%). The moderator analysis revealed that power to find the mean meta-analytic effect size did not moderate the effect of sleep deprivation on memory, *Q*(1) = 3.179, *p* = .075. All moderator analyses are reported in Table 4.[Fig fig9] [Table tbl4]

## Discussion

The two meta-analyses presented here aimed to quantify the size of the effect of sleep deprivation *after* learning and *before* learning on memory performance. Based on previous evidence for an effect of sleep on both declarative and procedural memories (Klinzing et al., 2019), we predicted that sleep deprivation would have a detrimental effect on learning and memory. We found that sleep deprivation after learning was associated with a mean effect size of *g* = 0.277. The effect size is positive, indicating that sleep deprivation has a detrimental rather than facilitatory impact on memory, as predicted by theory. Furthermore, the 95% confidence intervals around the mean do not cross zero, indicating that the effect size is statistically significantly higher than zero. For sleep deprivation before learning, we found an average effect size of *g* = 0.621. The effect size is positive, indicating that sleep deprivation before learning impairs rather than facilitates memory, as predicted by theory (e.g., Cirelli & Tononi, in press; Tononi & Cirelli, 2012). The 95% confidence intervals around the mean do not cross zero, again indicating that the effect size is statistically significantly higher than zero. Following Cohen’s guidelines for categorizing effect sizes a small (0.20), medium (0.50), and large (0.8), the effect sizes above would correspond to small-to-medium and medium. However, given that these cutoff points are wholly arbitrary and were only ever intended to be used as a last resort, many now argue that effect sizes should be interpreted in the context of typical effect sizes observed in the relevant literature (Correll et al., 2020; Funder & Ozer, 2019). According to Brysbaert (2019), an effect size of *d* = 0.40 represents an average effect size in experimental psychology *and* has practical and theoretical relevance. Putting our meta-analytic effect sizes into this context, it appears that sleep deprivation before learning has an effect size somewhat larger than the average effect size in experimental psychology, while sleep deprivation after learning has a somewhat smaller than average effect size, although the latter varies as a function of both recovery sleep and memory type (declarative vs. procedural), as discussed in detail below.

### Theory-Based Mediators

Despite the wide range of literature examining an effect of sleep deprivation on memory performance, this is, to our knowledge, the first time that the size of this effect has been formally quantified. A benefit of meta-analyses is that they allow for the investigation of potential moderating factors that may differentially influence the size of the meta-analytic effect. For deprivation after learning, we were able to investigate whether the first night of sleep is essential for the consolidation of newly acquired memories or whether a later sleep opportunity can compensate for the first night of sleep deprivation. We found that studies where memory was tested immediately after one night of sleep deprivation and before recovery sleep showed a significant sleep deprivation associated memory deficit (*g* = 0.410). Critically, those studies that had one or more nights of recovery sleep prior to retrieval also showed a small but statistically significant memory deficit (*g* = 0.176). Thus, memory impairments caused by sleep deprivation during the first postencoding night were still present but less severe when recovery sleep occurred before testing. On the one hand, this finding suggests that the first night of sleep after learning is important as its disruption is still felt even after recovery sleep. On the other hand, it also suggests that recovery sleep can to some extent mitigate the disruption of the first night of sleep by reducing the effect size by about 50%. While these data are consistent with theories arguing that, for hippocampal-dependent memories at least, the hippocampus may act as a buffer, retaining newly learned information until an offline consolidation opportunity is available (Schönauer et al., 2015), the idea that consolidation processes can be spread over multiple nights of sleep is yet to be explicitly tested.

It is also difficult to establish the extent to which the smaller effect of sleep deprivation after recovery sleep on memory is due to the occurrence of a delayed consolidation opportunity or due to effects of fatigue being diminished. Recent work has suggested that fatigue at time of test might have little or no detrimental impact on tasks assessing long-term memory. Schönauer et al. (2015) found that sleep deprivation before a recall task did not impair memory for previously encoded and consolidated word pairs. Furthermore, despite a difference in the size of the effect of sleep deprivation, it is important to note that we still see a significant detrimental effect of sleep deprivation after learning even when a later sleep opportunity is permitted, albeit a smaller effect. An account based on fatigue alone is insufficient to explain this finding. Another potential alternative explanation for the decrease in effect size after recovery sleep could be based on interference. When tested after one night of sleep or sleep deprivation, participants in the sleep group will have experienced little interference from subsequent cognitive activity after learning. A large difference between the groups at this point could be due to sleep protecting new memories from interference rather than due to active consolidation processes. After one or more nights of recovery sleep, both groups will have experienced some degree of interference, and this could explain the reduction in the effect size. Further research is needed to adjudicate between these different accounts that could both contribute to the effect sizes we have observed.

For deprivation after learning, we also found that whether the task type was declarative or procedural had an effect on the size of the deprivation effect. Although both declarative and procedural tasks elicited a significant effect, a moderator analysis indicated that those studies implementing procedural memory tasks had significantly larger effect sizes on average (*g* = .449) than declarative tasks (*g* = .218). That both declarative and procedural memory tasks showed detrimental effects of sleep deprivation was unsurprising, given that a benefit of sleep has been observed for both declarative memories (e.g., Gais & Born, 2004; Talamini et al., 2008; Wagner et al., 2006) and procedural memories (Korman et al., 2007; Schönauer et al., 2014; Walker et al., 2005). Our findings are also consistent with recent studies showing that sleep is beneficial even in tasks that do not require the hippocampus at learning (e.g., Schapiro et al., 2019). Although the current meta-analysis indicates a detrimental effect of sleep deprivation after learning on both declarative and procedural memories, the exact mechanisms that drive these effects are still debated, and thus it is unclear why procedural memories may show *larger* sleep deprivation effects. According to active systems consolidation theory, hippocampal-dependent declarative memories benefit from repeated reactivation of newly learned memories during sleep, supporting the strengthening of memory representations in the neocortex (Born & Wilhelm, 2012; Walker & Stickgold, 2006). However, procedural memories that rely on implicit learning are unlikely to be dependent on such hippocampal–neocortical representations. It has been theorized that such implicit memories require more immediate offline consolidation to see a beneficial effect of sleep (Schönauer et al., 2015; Stickgold et al., 2000). Thus, it may be that without an immediate sleep opportunity, the detrimental effects of sleep deprivation have a larger impact on procedural memory consolidation, whereas declarative memory consolidation is less impacted by the lack of an immediate sleep opportunity. However, we found no significant interaction between recovery sleep and task type, suggesting that for both declarative and procedural tasks, lack of an immediate sleep opportunity increased the size of the effect of sleep deprivation. Thus, recovery sleep had a similar impact on both procedural and declarative task performance, and procedural tasks elicited larger effect sizes than declarative tasks, regardless of whether recovery sleep occurred.

For deprivation after learning, we found no effect of emotional versus neutral memory on the size of the meta-analytic effect. Although some studies do suggest a preferential effect of sleep for emotional memories (e.g., Payne & Kensinger, 2010; Wagner et al., 2001), our findings join two recent meta-analyses that report no overall preferential effect of sleep on emotional memory consolidation (Lipinska et al., 2019; Schäfer et al., 2020). These existing meta-analyses focussed on emotional memory and were able to uncover mediators that may reveal boundary conditions for the preferential effect; however, the number of studies in this domain is still low and more research is needed to establish the reliability of the effect.

For both deprivation after learning and deprivation before learning, we found no effect of the recall versus recognition moderator on the size of the meta-analytic effect. This is in contrast to some previous studies investigating the beneficial role of sleep on memory that have found a differential effect of recall versus recognition testing, and in contrast to the meta-analysis of Newbury and Monaghan (2019), which looked at sleep studies using the DRM paradigm. Although performance on recall tasks repeatedly benefits from sleep, performance on recognition tasks has sometimes been found to show little or no offline consolidation benefit (Ashton et al., 2018; Diekelmann et al., 2009; Drosopoulos et al., 2005; Gais et al., 2006; Hu et al., 2006). It is posited that, although recall tasks rely on explicit, hippocampal-dependent memory, recognition tasks could include both an explicit recollection and implicit familiarity element (Jacoby, 1991), only the former of which benefits from sleep-associated consolidation. Thus, the mechanisms by which these two types of memories are consolidated may be different. Despite this, the present meta-analyses provide no evidence to suggest that performance on recall and recognition tasks are differentially affected by sleep deprivation either before or after sleep. Whether this finding extends to sleep paradigms other than total sleep deprivation remains to be established.

The null effects from our moderator analyses should be treated with caution, however, as we may not have adequate statistical power to detect smaller moderator effect sizes. Hempel et al. (2013) suggest that power to detect moderator effects is dependent on a combination of the amount of residual heterogeneity within the data set, the number of studies in the data set, the number of participants in the included studies, and the ratio of studies in the conditions compared against each other. Based on power simulations, Hempel et al. (2013) provide estimations of the approximate number of studies and participants required to detect categorical moderator effects of different effect sizes. We used data from these simulations to retrospectively assess the power of our moderator analyses to detect an effect. Since Hempel et al.’s simulations are not based on multilevel meta-analyses, the below estimates should be treated with caution when applied to our analyses and only considered as indicative.

For our deprivation after learning analysis, residual heterogeneity was τ^2^ = .026 for within-study variance, and τ^2^ = .061 for between-study variance. Therefore, based on a τ^2^ of between 0 and 0.1, the simulations suggest that the moderator recall versus recognition was powered to detect an effect of around 0.2–0.3 (based on 100 trials, 20 participants per study, at 80% power). The emotionality moderator was powered to detect only large moderator effects of 0.3–0.4 (based on 50 trials, 20 participants per study, 80% power). For the deprivation before learning analysis, residual heterogeneity was τ^2^ = .096 for between-study variance, and τ^2^ = .017 for within-study variance. The simulations suggest that the moderator analysis of recall versus recognition was powered to detect only a large effect size of between 0.3 and 0.4 (based on 50 trials, 20 participants per study, at 80% power). Therefore, it appears that our moderator analyses were not sufficiently powered to detect small moderator effects and the null findings in these analyses should be considered preliminary. These analyses need to be repeated as more evidence accumulates over time.

There are other moderators that would be valuable to account for to increase the precision of our meta-analytic effect size, but that we could not include in our analyses due to the small number of studies available. For example, some studies included in the meta-analysis involved manipulations that the authors expected to reverse or eradicate the detrimental effect of sleep deprivation. Kolibius et al. (2021) predicted that large amounts of encoded information (640 word pairs) would increase forgetting in the sleep group compared to a sleep-deprived group. Similarly, Feld et al. (2016) hypothesized that those in a high memory load condition (360 word pairs) should no longer show a sleep benefit compared to a sleep-deprived condition. Vargas et al. (2019) examined memory for emotionally negative and neutral objects and backgrounds, but only predicted an impact of sleep deprivation on neutral objects. It is possible that the inclusion of studies such as these (or conditions within those studies where no sleep deprivation effect is predicted) may have artificially reduced our meta-analytic effect size. A mediator analysis would be the appropriate solution to establish whether this was the case, but the small number of relevant studies prevents this for now.

### Quality-Based Moderators

The meta-analyses in this article suggest that there is a detrimental effect of sleep deprivation on learning and memory, and it is observed across a range of methodologies. However, our meta-analyses identified a number of potential limitations of the available data sets in this domain. Methodological quality scores ranged from 4 to 19 out of 22 in the studies investigating deprivation after learning; and they ranged from 7 to 19 in the studies investigating deprivation before learning.

We must be cautious in the way that we interpret the effects of methodological quality on the size of the effect of sleep deprivation. Valentine (2009) argues that methodological scales of this nature frequently lack operational specificity (e.g., that each item deserves equal weight) and include questions that are unclear. In an attempt to increase the validity of our methodological quality checklist, we designed our checklist based on the Downs and Black checklist (1998), with modified questions relevant to sleep studies. For the first meta-analysis, we found a significant mediating effect of the reporting cluster of our methodological quality checklist on the size of the sleep deprivation effect, Figure 4 shows that studies scoring highest on this cluster show no effect of sleep deprivation while the lower scoring studies do. The items in the reporting cluster are predominantly concerned with the number and nature of exclusion and inclusion criteria used in the study. It therefore appears that the studies showing higher effect sizes may have employed fewer such criteria. However, some of the scores on this cluster may be underestimated due to incomplete reporting. For example, studies stating that they only recruited healthy participants may have used other sleep-related inclusion and exclusion criteria, such as excluding participants who were taking medication that affects sleep or those who had recently traveled between time zones, without reporting these and may have scored higher on this cluster had these criteria been reported. We found no mediating effect of any cluster of methodological quality on the size of the effect of sleep deprivation for the second meta-analysis. Given that only one cluster of the quality score influenced the size of the effect in the first meta-analysis, and no clusters had a significant effect in the second meta-analysis, our effect size estimates are unlikely to be substantially biased by variation in methodological quality.

Taking a broader qualitative view of our quality checklist, we note that only one of the analyzed studies was preregistered, and only three justified their sample size with an a priori power analysis. Given that preregistration has become a mainstream practice only in the past few years (Nosek & Lindsay, 2018), and that an a priori power analysis is part of the preregistration process, the low numbers here are unsurprising and are likely in line with the current broader field of experimental psychology. The key quality measures on study design were met by the clear majority of studies (e.g., equal group sizes, random allocation to groups or counterbalancing of conditions).

### Power-Based Moderators

In the current meta-analyses, we calculated statistical power to find the meta-analytic effect size for each experiment and assessed whether statistical power significantly influenced the size of the effect of sleep deprivation. For sleep deprivation after learning, mean statistical power to find the meta-analytic effect size was just 14%; for sleep deprivation before learning, it was higher though still far less than optimal at 55%. Given that power is a function of the effect size, sample size, and the statistical test being employed, the difference in obtained power across the two research questions is understandable: As the effect size decreases, power to detect it decreases if sample size is held constant. Overall, these figures are closely in line with the broader field: For example, Szucs and Ioannidis (2017) found that within psychology and cognitive neuroscience, mean power to detect small, medium, and large effects (in Cohen’s terms) was 17%, 49%, and 71%, respectively.

Given the current convention that statistical power to find an effect is at 80% or higher (Di Stefano, 2003), it is evident that the majority of the studies in these meta-analyses are underpowered. This is problematic as it increases the uncertainty around our meta-analytical effect sizes. To better understand the consequences of the uncertainty introduced by low power in the studies included in our meta-analyses, we investigated whether statistical power to find the mean meta-analytic effect size influenced the size of the sleep deprivation effect by entering obtained power as a moderator. For example, it might be the case that it is only low-powered studies that show an impact of sleep deprivation, while high-powered studies might show no impact. Such a pattern would suggest that our meta-analytic effect size might be overestimated as a consequence of low power. The opposite pattern would suggest that our effect size has been underestimated due to low power. For deprivation after learning, we found no moderating impact of statistical power on the size of the effect. In other words, both low- and high-powered studies yielded similar effect sizes. However, the validity of this analysis is reduced by the fact that there were no studies in this meta-analysis where power exceeded 33%, and therefore we have no way of knowing what effect sizes could be expected when power is higher. For deprivation before learning, we found a broader range of power extending from about 20% to over 90%. However, once again we found no statistically significant moderating impact of power on the size of the effect.

To gain a more precise estimate of the true effect size, future studies should use the current meta-analytic effect size as a guide in determining sample sizes that will yield high power. Studies planning to look at sleep deprivation after learning and running a two-tailed *t*-test for a between-subjects design with a sleep versus sleep deprivation manipulation would require a sample size of approximately 410 to have 80% power to detect the meta-analytic effect size. For a within-subjects design, the sample size required would be 105. For studies examining deprivation before learning, a two-tailed *t*-test with a between-subjects design would require a sample size of 82, whereas a within-subjects design would require a much smaller sample size of 23, to have 80% power to detect the meta-analytic effect size. The above numbers are rough indications only, and lower or higher sample sizes may be appropriate depending on the specific design of the experiment and the statistical analysis approach (Brysbaert, 2019; Lakens & Caldwell, 2021).

It is clear that there is a significant discrepancy between the high-power sample sizes we have estimated above and the sample sizes found in the majority of the studies included in the current meta-analyses. This discrepancy is important as there are severe limitations to the strength of conclusions that can be drawn from underpowered individual studies (see, e.g., Brysbaert, 2019, for a detailed discussion). Fraley and Vazire (2014) described three limitations: (a) underpowered studies are less likely than properly powered studies to detect a true effect; (b) underpowered studies are more likely to yield false-positive findings than properly powered studies; and (c) underpowered studies are less likely than properly powered studies to produce replicable findings. Wilson et al. (2020) further demonstrate that underpowered studies are likely to yield inflated effect sizes. Therefore, the results of any single underpowered study should be treated with caution, and a meta-analytic approach such as ours may be the more useful approach for extracting information from these studies. Yet, small-scale studies are not always completely uninformative; we return to this debate in the Conclusions section.

### Publication Bias

Conducting a meta-analysis allows for an estimation of publication bias within the literature. Publication bias is evident when there are a large number of published studies in the direction of the hypothesis, with few nonsignificant published studies (Rosenthal, 1979). This can lead to overestimation of the size of the effect. We found statistically significant evidence of publication bias in both meta-analyses. Adjusting the deprivation after learning effect size for publication bias using the trim-and-fill method changed the effect size from 0.277 to 0.166, and changed the deprivation before learning effect size from 0.621 to 0.463, although these adjusted effect size should be treated with caution given that the trim-and-fill method was not designed for a multilevel approach. Nonetheless, both estimates remained significantly different from zero after the adjustment. To allow for more accurate effect size estimates in future meta-analyses, we suggest researchers in this field should adopt registered reports as an effective way of ensuring all results find their way into published literature.

### Limitations

We focused specifically on the effects of total (overnight) sleep deprivation, and thus future meta-analyses are needed to establish whether the effect size is similar in studies using sleep restriction. We chose to concentrate on studies using total sleep deprivation because depriving a participant of all sleep is a stronger test of the hypothesis that sleep benefits memory than depriving them of a single stage of sleep or restricting their sleep for some hours over a period of time, as discussed in the Introduction section. An alternative approach could have been to include restriction studies and to conduct a moderator analysis to establish whether they lead to similar effect sizes as total deprivation. However, many sleep restriction studies in the literature are field studies that lack the rigorous controls we include in our inclusion criteria (e.g., lack of control over hours slept, Deary & Tait, 1987; inappropriate sleep control condition, Piérard et al., 2004), and therefore the number of eligible restriction studies would have been smaller than the number of total deprivation studies. As discussed earlier, such imbalance in number of studies can make moderator analyses insensitive (Hempel et al., 2013).

Our search focused solely on English language reports, thus risking a mono-language bias (Johnson, 2021). This restricts our ability to generalize the results of our meta-analyses to non-English language literature. In particular, by using English language sources only, there is the possibility that our search missed much of the gray literature such as PhD theses and conference abstracts written in other languages. The use of solely English language sources limits our understanding of any possible cross-cultural differences in effects of sleep on memory. Indeed, there are many cross-cultural differences in sleep habits (e.g., Cheung et al., 2021), and although we are not aware of any studies that have systematically compared sleep-associated memory consolidation effects across cultures, our reliance on English language literature means that we would not have captured such differences if they do exist.

We acknowledge that our inclusion criteria restrict our ability to draw conclusions beyond healthy, typical populations. We excluded studies that included participants under the age of 18 and studies that involved participants suffering from sleep disorders or psychiatric disorders. There is growing interest in understanding how sleep-associated memory consolidation in these groups might differ from healthy adults, however (e.g., Hoedlmoser, 2020; Manoach & Stickgold, 2019), and future meta-analyses addressing these questions will be valuable both for theoretical development and practical reasons. Finally, we note that there were 17 studies that we were unable to include in the analyses as the required statistical information was not reported (see Figure 1); unfortunately, the authors of these papers were unable to provide with the necessary data when contacted. Nonetheless, these studies made up a small proportion of the studies we identified as eligible and would be unlikely to change the conclusions we have drawn.

## Conclusions

To conclude, the two meta-analyses presented here provide a comprehensive analysis of the impact of sleep deprivation after learning and before learning. The results of the first meta-analysis suggest that depriving participants of the first night of sleep after encoding new information results in lower performance at test, supporting the theories of sleep-associated memory consolidation (e.g., Diekelmann & Born, 2010; McClelland et al., 1995). This effect was larger before than after recovery sleep and larger in procedural memory tasks compared to declarative memory tasks. The results of the second meta-analysis suggest that sleep-deprived participants are able to encode less information than rested controls, supporting the theories that propose that sleep restores memory encoding capacity (e.g., Saletin & Walker, 2012; Tononi & Cirelli, 2012).

We found that levels of statistical power tended to be low, particularly in those studies looking at sleep deprivation after learning in which there was a small estimated effect size. Given that underpowered studies are ubiquitous across disciplines that use human participants (Dumas-Mallet et al., 2017), new ways of interpreting low-powered studies are emerging. One particularly insightful interpretation has recently been offered by Wilson et al. (2020). In short, Wilson and colleagues draw a distinction between “original science” and “replication science.” Original science is roughly science as practiced today, in that it uses Null Hypothesis Significance Testing combined with study designs whose power falls far short of the gold standard of high-N studies. Original science in this formulation serves an important and inexpensive screening function to identify effects that may be true and would therefore benefit from further, more costly examination of replication science. Replication science consists of high-N, highly powered, and costly direct replications of the key studies from original science, vital for verifying the preliminary results of original science. Applying this framework to the literature targeted in our meta-analyses, we propose that there is now sufficient evidence from original science to warrant a move to replication science in this field. No highly powered, preregistered direct replications looking at the role of sleep deprivation in learning and memory have been conducted thus far. The meta-analytic estimates of the relevant effect sizes provided here will facilitate the design of such urgently needed studies, while also allowing better informed sample size choice for continuing original science efforts.

## Supplementary Material

10.1037/bul0000348.supp

## Figures and Tables

**Table 1 tbl1:** Features of Included Sleep Deprivation After Learning Interventions (k = 130)

Study feature	*k*	*M* ± *SD*	*Mdn*	Mode	Range
Publication date	130	NA	2012	2015	1994–2020
Sample size	130	31.84 ± 13.15	28	28	6–78
Age	54	22.15 ± 1.41	22.30	23.30	18.10–24.30
% Females	95	56.37 ± 19.04	56.25	50.00	0.00–85.71
Study design	130				
Between-groups	104	NA	NA	NA	NA
Within-group	26	NA	NA	NA	NA
Paradigm	130				
Motor skill	15	NA	NA	NA	NA
Recognition	60	NA	NA	NA	NA
Recall	42	NA	NA	NA	NA
Route learning	4	NA	NA	NA	NA
Temporal order	2	NA	NA	NA	NA
Mere exposure effect	1	NA	NA	NA	NA
Categorization	6	NA	NA	NA	NA
Stimuli	130				
Words	36	NA	NA	NA	NA
Scenes	30	NA	NA	NA	NA
Images	47	NA	NA	NA	NA
Sequence	15	NA	NA	NA	NA
Instruction	1	NA	NA	NA	NA
Trajectory	1	NA	NA	NA	NA
Emotionality of stimuli	130				
Neutral	20	NA	NA	NA	NA
Emotional	31	NA	NA	NA	NA
Not reported	79	NA	NA	NA	NA
Memory type	130				
Procedural	21	NA	NA	NA	NA
Declarative	109	NA	NA	NA	NA
Sleep deprivation compliance check	130				
Human observation of night	107	NA	NA	NA	NA
Human observation of day and night	12	NA	NA	NA	NA
No human observation	11	NA	NA	NA	NA
Recovery sleep	130				
Yes	85	NA	NA	NA	NA
No	45	NA	NA	NA	NA
Nights recovery sleep	74^a^	3.18 ± 3.02	2.00	2.00	1–13
Statistical power to detect meta-analytic effect size^b^	130	13.95% ± 5.00	13%	11%	7%–30%
Quality—reporting	130	0.56 ± 0.21	0.50	0.50	0.00–1.00
Quality—bias	130	0.74 ± 0.19	0.71	0.71	0.29–1.00
Quality—confounding	130	0.77 ± 0.15	0.83	0.83	0.33–1.00
^a^ Ten effect sizes had 6 months of recovery sleep and were excluded from the table as outliers. One study reported one to six nights of recovery sleep and was excluded from the table due to lack of precision. ^b^ Statistical power to detect the meta-analytic effect size of Hedges’ *g* = 0.277, with α at .05.					

**Table 2 tbl2:** Effect of Each Moderator on the Overall Meta-Analytic Effect of Sleep Deprivation After Learning on Memory Performance

Moderator	Variable type	*df*	Heterogeneity (*Q*)	*p*
Recovery sleep (yes vs. no)	Categorical	1	10.496	<.001*
Task type (declarative vs. procedural)	Categorical	1	5.301	.021*
Recall versus recognition	Categorical	1	0.115	.734
Emotionality (emotional vs. neutral)	Categorical	1	0.169	.681
Quality—reporting cluster	Continuous	1	9.337	.002*
Quality—bias cluster	Continuous	1	0.989	.320
Quality—confounding cluster	Continuous	1	0.049	.825
Power	Continuous	1	0.611	.434
Recovery sleep × Task type	Categorical	1	0.804	.370
* *p* < .05.				

**Table 3 tbl3:** Features of Included Sleep Deprivation Before Learning Interventions (k = 55)

Study feature	*k*	*M*	*Mdn*	Mode	Range
Publication date	55	NA	2010	2000/2020	1989–2020
Sample size	55	30.75 ± 13.82	26	26	12–58
Age	27	24.55 ± 5.89	22.19	20.70	19.50–47.83
% Female	42	47.78 ± 22.16	50.00	50.00	00.00–74.36
Study design	55				
Between-groups	34	NA	NA	NA	NA
Within-group	21	NA	NA	NA	NA
Paradigm	55				
Recognition	23	NA	NA	NA	NA
Cued recall	14	NA	NA	NA	NA
Free recall	12	NA	NA	NA	NA
Texture discrimination	1	NA	NA	NA	NA
Recency discrimination	2	NA	NA	NA	NA
Finger tapping	1	NA	NA	NA	NA
Categorization	2	NA	NA	NA	NA
Stimuli	55				
Words	24	NA	NA	NA	NA
Images	23	NA	NA	NA	NA
Prose	3	NA	NA	NA	NA
Numbers	1	NA	NA	NA	NA
Instruction	4	NA	NA	NA	NA
Memory type	55				
Procedural	2	NA	NA	NA	NA
Declarative	53	NA	NA	NA	NA
Sleep deprivation compliance	55				
Human observation of night	42	NA	NA	NA	NA
Human observation of day and night	10	NA	NA	NA	NA
No human observation	3	NA	NA	NA	NA
Recovery sleep	55				
Yes	13	NA	NA	NA	NA
No	42	NA	NA	NA	NA
Nights recovery sleep	12^a^	6.04	2.00	2.00	2–13
Statistical power to detect meta-analytic effect size^b^	55	54.77 ± 20.79	54	86	21–98
Quality—reporting	55	0.61 ± 0.28	0.50	0.50	0.00–1.00
Quality—bias	55	0.71 ± 0.23	0.71	0.86	0.14–1.00
Quality—confounding	55	0.81 ± 0.16	0.83	0.83	0.50–1.00
^a^ One study using recovery sleep only had a 90-min nap opportunity as recovery sleep and was excluded from the table as an outlier. ^b^ Statistical power to detect the meta-analytic effect size of Hedges’ *g* = 0.621, with α at .05.					

**Table 4 tbl4:** Effect of Each Moderator on the Overall Meta-Analytic Effect of Sleep Deprivation Before Learning on Memory Performance

Moderator	Variable type	*df*	Heterogeneity (*Q*)	*p*
Recall versus recognition	Categorical	1	0.261	.610
Quality—reporting cluster	Continuous	1	0.868	.351
Quality—bias cluster	Continuous	1	0.064	.800
Quality—confounding cluster	Continuous	1	0.867	.352
Power	Continuous	1	3.110	.078
