# Supplementary material for: Sleep Deprivation and Memory: Meta-Analytic Reviews of Studies on Sleep Deprivation Before and After Learning
Source: Psychol Bull. 2021 Nov;147(11):1215–40. doi: 10.1037/bul0000348 (PMC8893218; doi:10.1037/bul0000348)
Supplement: Supplementary file 1 [file BUL-2020-0254_Supplemental_Materials.docx]

Appendix A

**Methodological** **Quality Checklist**

*Reporting*

1. Did the study exclude participants with neurological disorders?
2. Did the study exclude participants with psychiatric disorders?
3. Did the study exclude participants with a history of sleep disorders?
4. Did the study exclude participants taking medication that affects sleep?
5. Did the study exclude participants who had travelled across time zones in the last four weeks?
6. For between-group studies, were the deprivation and control groups matched for chronotype? (Within-group studies automatically score 1)

*Bias*

1. Was the study pre-registered?
2. Was optional stopping in data collection excluded through a priori justification of sample size or through other measures such as interim data analysis with appropriate controls for Type 1 error rate?
3. Was the time of training and test the same between control and sleep deprivation groups?
4. Were instructions given, and checked via sleep diaries / actigraphy / direct observation, for pre-experimental sleep?
5. Were the control group given in-bed and out-of-bed instructions?
6. Were the sleep circumstances of the control group monitored using actigraphy, sleep diaries, or direct observation?
7. Was interference for the sleep deprivation group low (non-demanding activities given and monitored in the lab?)
8. Was the sleep deprivation group kept under continuous human observation throughout the period of sleep deprivation, including night and day times (i.e to stop them napping throughout the day)?
9. Was the sleep deprivation group kept under continuous human observation throughout the night (excludes actigraphy and sleep diaries because they do not ensure compliance)?

*Confounding*

1. Was the study a within-group design (this is referring to the deprivation/control factor)?
2. For within-group studies, was the order of deprivation and control conditions counterbalanced? (Between group studies automatically score 1)
3. For between-group studies, was there random allocation to the deprivation and control groups? (Within group studies automatically score 1)
4. Were the deprivation and control groups treated similarly except the primary manipulation?
5. Was there sleep inertia recovery time for the control group?
6. For between-group studies, were the sample sizes for the deprivation and control groups equal, or with a difference that was <15% of the overall sample size?

*Power*

1. Did the study report an a priori power analysis with power set at 80% or higher, and alpha at 0.05 or lower?

Appendix B

| Summary of studies investigating the effect of sleep deprivation after learning on memory | | | | |  |  |  | |  | |
| --- | --- | --- | --- | --- | --- | --- | --- | --- | --- | --- |
| Citation^1^ | N | Study Design | Paradigm*^2^ | Memory Type* | Recovery sleep before test session?* | Power^3^* | Effect Size | | Emotionality* | |
| **Albouy et al. (2013b)** | 30 | Between-groups | Motor Skill | Procedural | Yes | 11 | 0.280 | | None | |
| **Ashton et al. (2020);** Experiment 2, Test 1 - Negative Stimuli | 28 | Within-group | Recognition | Declarative | No | 29 | 0.457 | | Emotional | |
| **Ashton et al. (2020);** Experiment 2, Test 1 - Neutral Stimuli | 28 | Within-group | Recognition | Declarative | No | 29 | 0.506 | | Neutral | |
| **Ashton et al. (2020);** Experiment 2, Test 2 - Negative Stimuli | 28 | Within-group | Recognition | Declarative | Yes | 29 | 0.188 | | Emotional | |
| **Ashton et al. (2020);** Experiment 2, Test 2 - Neutral Stimuli | 28 | Within-group | Recognition | Declarative | Yes | 29 | 0.386 | | Neutral | |
| **Atienza & Cantero (2008);** Neutral Stimuli – Remember Judgements | 28 | Between-groups | Recognition | Declarative | Yes | 11 | 0.900 | | Neutral | |
| **Atienza & Cantero (2008);** Emotional Stimuli – Remember Judgements | 28 | Between-groups | Recognition | Declarative | Yes | 11 | 0.896 | | Emotional | |
| **Atienza & Cantero (2008);** Neutral Stimuli – Know Judgements | 28 | Between-groups | Recognition | Declarative | Yes | 11 | -0.719 | | Neutral | |
| **Atienza & Cantero (2008);** Emotional Stimuli – Know Judgements | 28 | Between-groups | Recognition | Declarative | Yes | 11 | -0.603 | | Emotional | |
| **Borragán et al. (2015)** | 33 | Between-groups | Motor Skill | Procedural | Yes | 12 | 0.482 | | None | |
| **Darsaud et al. (2011);** Know judgements | 36 | Between-groups | Recognition | Declarative | Yes | 13 | -1.064 | | None | |
| **Darsaud et al. (2011);** Remember judgements | 36 | Between-groups | Recognition | Declarative | Yes | 13 | 0.872 | | None | |
| **Deliens et al. (2013a)** | 22 | Within-group | Recall | Declarative | Yes | 24 | -0.491 | | None | |
| **Diekelmann et al. (2008);** Experiment 1 | 29 | Between-groups | Recognition | Declarative | No | 11 | -0.507 | | None | |
| **Diekelmann et al. (2008);** Experiment 3 | 32 | Between-groups | Recognition | Declarative | Yes | 12 | -0.405 | | None | |
| **Diekelmann et al. (2010);** High Performers | 16 | Between-groups | Recall | Declarative | No | 8 | 0.539 | | None | |
| **Diekelmann et al. (2010);** Low Performers | 21 | Between-groups | Recall | Declarative | No | 9 | 0.059 | | None | |
| **Diekelmann et al. (2013);** Experiment 1 | 35 | Between-groups | Recall | Declarative | Yes | 13 | 1.087 | | None | |
| **Feld et al. (2016);** Experiment 1 - 160 Word Pairs | 48 | Between-groups | Recall | Declarative | Yes | 16 | 0.837 | | None | |
| **Feld et al. (2016);** Experiment 1 - 40 Word Pairs | 48 | Between-groups | Recall | Declarative | Yes | 16 | 0.378 | | None | |
| **Feld et al. (2016);** Exepriment 2 - 320 Word Pairs | 53 | Between-groups | Recall | Declarative | Yes | 17 | 0.111 | | None | |
| **Feld et al. (2016);** Exepriment 2 - 40 Word Pairs | 53 | Between-groups | Recall | Declarative | Yes | 17 | 0.162 | | None | |
| **Ferrara et al. (2006)** | 34 | Between-groups | Route Learning | Procedural | No | 12 | 0.808 | | None | |
| **Ferrara et al. (2008);** Groups 1 & 2 | 20 | Between-groups | Route Learning | Procedural | No | 9 | 0.470 | | None | |
| **Ferrara et al. (2008);** Group 5 & 6 | 20 | Between-groups | Route Learning | Procedural | Yes | 9 | 0.895 | | None | |
| **Fischer et al. (2002)** | 20 | Between-groups | Motor Skill | Procedural | No | 9 | 1.636 | | None | |
| **Fischer et al. (2002);** Supplementary Experiment | 6 | Within-group | Motor Skill | Procedural | Yes | 9 | 0.249 | | None | |
| **Fischer et al. (2005)** | 8 | Within-group | Motor Skill | Procedural | Yes | 11 | 0.549 | | None | |
| **Fischer et al. (2005);** Supplementary Experiment | 10 | Within-group | Motor Skill | Procedural | No | 12 | 0.422 | | None | |
| **Gais et al. (2006);** Experiment B | 14 | Within-group | Recall | Declarative | Yes | 16 | 0.671 | | None | |
| **Griessenberger et al. (2012);** Test 1 | 34 | Between-groups | Temporal Order | Declarative | No | 12 | 1.037 | | None | |
| **Griessenberger et al. (2012);** Test 2 | 34 | Between-groups | Temporal Order | Declarative | Yes | 12 | 0.721 | | None | |
| **Harrington et al. (2018);** Test 1, High BDI-II - Negative Images | 26 | Between-groups | Recognition | Declarative | No | 10 | -0.141 | | Emotional | |
| **Harrington et al. (2018);** Test 1, High BDI-II - Neutral Images | 26 | Between-groups | Recognition | Declarative | No | 10 | 0.256 | | Neutral | |
| **Harrington et al. (2018);** Test 1, High BDI-II - Positive Images | 26 | Between-groups | Recognition | Declarative | No | 10 | -0.224 | | Emotional | |
| **Harrington et al. (2018);** Test 1, Low BDI-II - Negative Images | 28 | Between-groups | Recognition | Declarative | No | 11 | -0.225 | | Emotional | |
| **Harrington et al. (2018);** Test 1, Low BDI-II - Neutral Images | 28 | Between-groups | Recognition | Declarative | No | 11 | 0.067 | | Neutral | |
| **Harrington et al. (2018);** Test 1, Low BDI-II - Positive Images | 28 | Between-groups | Recognition | Declarative | No | 11 | 0.403 | | Emotional | |
| **Harrington et al. (2018);** Test 2, High BDI-II - Negative Images | 26 | Between-groups | Recognition | Declarative | Yes | 10 | -0.488 | | Emotional | |
| **Harrington et al. (2018);** Test 2, High BDI-II - Neutral Images | 26 | Between-groups | Recognition | Declarative | Yes | 10 | -0.011 | | Neutral | |
| **Harrington et al. (2018);** Test 2, High BDI-II - Positive Images | 26 | Between-groups | Recognition | Declarative | Yes | 10 | -0.009 | | Emotional | |
| **Harrington et al. (2018);** Test 2, Low BDI-II - Negative Images | 28 | Between-groups | Recognition | Declarative | Yes | 11 | -0.880 | | Emotional | |
| **Harrington et al. (2018);** Test 2, Low BDI-II - Neutral Images | 28 | Between-groups | Recognition | Declarative | Yes | 11 | -0.543 | | Neutral | |
| **Harrington et al. (2018);** Test 2, Low BDI-II - Positive Images | 28 | Between-groups | Recognition | Declarative | Yes | 11 | -0.154 | | Emotional | |
| **Idzikowski (1984);** Experiment 1 | 12 | Between-groups | Recall | Declarative | No | 7 | 0.753 | | None | |
| **Kuriyama et al. (2010);** Test 1 - MVA Stimuli | 28 | Between-groups | Recognition | Declarative | Yes | 11 | 0.230 | | Emotional | |
| **Kuriyama et al. (2010);** Test 1 - Safe Stimuli | 28 | Between-groups | Recognition | Declarative | Yes | 11 | 0.052 | | Neutral | |
| **Kuriyama et al. (2010);** Test 2 - MVA Stimuli | 28 | Between-groups | Recognition | Declarative | Yes | 11 | 0.387 | | Emotional | |
| **Kuriyama et al. (2010);** Test 2 - Safe Stimuli | 28 | Between-groups | Recognition | Declarative | Yes | 11 | 0.216 | | Neutral | |
| **Kuriyama et al. (2013);** Directed Remembering - MVA Stimuli | 31 | Between-groups | Recognition | Declarative | Yes | 11 | 0.282 | | Emotional | |
| **Kuriyama et al. (2013);** Directed Remembering - Safe Stimuli | 31 | Between-groups | Recognition | Declarative | Yes | 11 | 0.248 | | Neutral | |
| **Lahl et al. (2006);** Word-pair Recall | 24 | Between-groups | Recall | Declarative | No | 10 | 0.223 | | None | |
| **Lahl et al. (2006);** Word-Pair Recognition | 24 | Between-groups | Recognition | Declarative | No | 10 | -0.063 | | None | |
| **Lin et al. (2014)** | 30 | Between-groups | Recognition | Declarative | Yes | 11 | 0.800 | | None | |
| **Mograss et al. (2009);** New Items | 18 | Within-group | Recognition | Declarative | No | 20 | 0.474 | | None | |
| **Mograss et al. (2009);** Old Items | 18 | Within-group | Recognition | Declarative | No | 20 | 0.171 | | None | |
| **Nesca et al. (1994);** Experiment 2 | 48 | Between-groups | Recognition | Declarative | No | 16 | 0.521 | | None | |
| **Orban et al. (2006)** | 22 | Between-groups | Route Learning | Procedural | Yes | 10 | 0.379 | | None | |
| **Peigneux et al. (2007);** Left Hemisphere - MASK | 45 | Between-groups | Mere Exposure Effect | Declarative | Yes | 15 | 0.511 | | None | |
| **Peigneux et al. (2007);** Right Hemisphere - MASK | 45 | Between-groups | Mere Exposure Effect | Declarative | Yes | 15 | -0.369 | | None | |
| **Peigneux et al. (2007);** Left Hemisphere - MIRROR | 45 | Between-groups | Mere Exposure Effect | Declarative | Yes | 15 | 0.438 | | None | |
| **Peigneux et al. (2007);** Right Hemisphere - MIRROR | 45 | Between-groups | Mere Exposure Effect | Declarative | Yes | 15 | 0.477 | | None | |
| **Peigneux et al. (2007);** Left Hemisphere - SAME | 45 | Between-groups | Mere Exposure Effect | Declarative | Yes | 15 | 0.159 | | None | |
| **Peigneux et al. (2007);** Right Hemisphere - SAME | 45 | Between-groups | Mere Exposure Effect | Declarative | Yes | 15 | 0.610 | | None | |
| **Porcheret et al. (2015);** Intrusive Memory Recall | 42 | Between-groups | Recall | Declarative | Yes | 14 | 0.461 | | None | |
| **Schönauer et al. (2015);** Experiment 1, Test 1 - Mirror-Tracing | 21 | Within-group | Motor Skill | Procedural | No | 23 | 0.479 | | None | |
| **Schönauer et al. (2015);** Experiment 1, Test 1 - Word-Pair Recall | 21 | Within-group | Recall | Declarative | No | 23 | 0.631 | | None | |
| **Schönauer et al. (2015);** Experiment 1, Test 2 - Mirror-Tracing | 21 | Within-group | Motor Skill | Procedural | Yes | 23 | 0.583 | | None | |
| **Schönauer et al. (2015);** Experiment 1, Test 2 - Word-Pair Recall | 21 | Within-group | Recall | Declarative | Yes | 23 | -0.159 | | None | |
| **Schönauer et al. (2015);** Experiment 2, Sequential Finger-Tapping - Group A | 12 | Within-group | Motor Skill | Procedural | No | 14 | 0.195 | | None | |
| **Schönauer et al. (2015);** Experiment 2, Verbal Recall - Group A | 12 | Within-group | Recall | Declarative | No | 14 | 0.551 | | None | |
| **Schönauer et al. (2015);** Experiment 2, Sequential Finger-Tapping - Group B | 12 | Within-group | Motor Skill | Procedural | Yes | 14 | -0.015 | | None | |
| **Schönauer et al. (2015);** Experiment 2, Verbal Recall - Group B | 12 | Within-group | Recall | Declarative | Yes | 14 | -0.073 | | None | |
| **Schönauer et al. (2015);** Experiment 2, Sequential Finger-Tapping - Group C | 12 | Within-group | Motor Skill | Procedural | Yes | 14 | 0.242 | | None | |
| **Schönauer et al. (2015);** Experiment 2, Verbal Recall - Group C | 12 | Within-group | Recall | Declarative | Yes | 14 | -0.177 | | None | |
| **Sterpenich et al. (2007);** Negative Stimuli – Remember Judgements | 40 | Between-groups | Recognition | Declarative | Yes | 14 | 0.005 | | Emotional | |
| **Sterpenich et al. (2007);** Neutral Stimuli – Remember Judgements | 40 | Between-groups | Recognition | Declarative | Yes | 14 | 0.291 | | Neutral | |
| **Sterpenich et al. (2007);** Positive Stimuli – Remember Judgements | 40 | Between-groups | Recognition | Declarative | Yes | 14 | 0.595 | | Emotional | |
| **Sterpenich et al. (2007);** Negative Stimuli – Know Judgements | 40 | Between-groups | Recognition | Declarative | Yes | 14 | 0.091 | | Emotional | |
| **Sterpenich et al. (2007);** Neutral Stimuli – Know Judgements | 40 | Between-groups | Recognition | Declarative | Yes | 14 | -0.060 | | Neutral | |
| **Sterpenich et al. (2007);** Positive Stimuli – Know Judgements | 40 | Between-groups | Recognition | Declarative | Yes | 14 | -0.300 | | Emotional | |
| **Sterpenich et al. (2009);** Negative Stimuli – Remember Judgements | 33 | Between-groups | Recognition | Declarative | Yes | 12 | 0.188 | | Emotional | |
| **Sterpenich et al. (2009);** Neutral Stimuli – Remember Judgements | 33 | Between-groups | Recognition | Declarative | Yes | 12 | 0.426 | | Neutral | |
| **Sterpenich et al. (2009);** Positive Stimuli – Remember Judgements | 33 | Between-groups | Recognition | Declarative | Yes | 12 | 0.490 | | Emotional | |
| **Sterpenich et al. (2009);** Negative Stimuli – Know Judgements | 33 | Between-groups | Recognition | Declarative | Yes | 12 | -0.088 | | Emotional | |
| **Sterpenich et al. (2009);** Neutral Stimuli – Know Judgements | 33 | Between-groups | Recognition | Declarative | Yes | 12 | -0.380 | | Neutral | |
| **Sterpenich et al. (2009);** Positive Stimuli – Know Judgements | 33 | Between-groups | Recognition | Declarative | Yes | 12 | -0.081 | | Emotional | |
| **Sterpenich et al. (2017);** Recognition Task - Remote Words | 30 | Between-groups | Recognition | Declarative | Yes | 11 | -0.493 | | None | |
| **Tempesta et al. (2015);** Negative Stimuli | 54 | Between-groups | Recognition | Declarative | No | 17 | 0.598 | | Emotional | |
| **Tempesta et al. (2015);** Neutral Stimuli | 54 | Between-groups | Recognition | Declarative | No | 17 | 0.375 | | Neutral | |
| **Tempesta et al. (2015);** Positive Stimuli | 54 | Between-groups | Recognition | Declarative | No | 17 | 0.233 | | Emotional | |
| **Tempesta et al. (2017)** | 48 | Between-groups | Recognition | Declarative | Yes | 16 | 0.622 | | None | |
| **van Heughten et al. (2015);** Free Recall Task, Negative Stimuli - Rater 1 | 56 | Between-groups | Recall | Declarative | No | 18 | 0.513 | | Emotional | |
| **van Heughten et al. (2015);** Free Recall Task, Negative Stimuli - Rater 2 | 56 | Between-groups | Recall | Declarative | No | 18 | 0.197 | | Emotional | |
| **van Heughten et al. (2015);** Free Recall Task, Neutral Stimuli - Rater 1 | 56 | Between-groups | Recall | Declarative | No | 18 | 0.665 | | Neutral | |
| **van Heughten et al. (2015);** Free Recall Task, Neutral Stimuli - Rater 2 | 56 | Between-groups | Recall | Declarative | No | 18 | 0.252 | | Neutral | |
| **van Heughten et al. (2015);** Free Recall Task, Positive Stimuli - Rater 1 | 56 | Between-groups | Recall | Declarative | No | 18 | 0.542 | | Emotional | |
| **van Heughten et al. (2015);** Free Recall Task, Positive Stimuli - Rater 2 | 56 | Between-groups | Recall | Declarative | No | 18 | 0.574 | | Emotional | |
| **Vargas et al. (2019);** Neutral Object | 39 | Between-groups | Recognition | Declarative | No | 13 | 0.621 | | Neutral | |
| **Vargas et al. (2019);** Neutral Background | 39 | Between-groups | Recognition | Declarative | No | 13 | 0.434 | | Neutral | |
| **Vargas et al. (2019);** Negative Object | 39 | Between-groups | Recognition | Declarative | No | 13 | 0.310 | | Emotional | |
| **Vargas et al. (2019);** Negative Background | 39 | Between-groups | Recognition | Declarative | No | 13 | 0.706 | | Emotional | |
| **Montemayor (2014);** Unpublished | 30 | Between-groups | Motor Skill | Procedural | No | 11 | 0.817 | | None | |
| **Tamminen et al. (2020);** Old-New Decision Task - Test 1 | 46 | Between-groups | Recognition | Declarative | Yes | 15 | -0.422 | | None | |
| **Tamminen et al. (2020);** Reading Aloud Task - Test 1 | 46 | Between-groups | Recall | Declarative | Yes | 15 | -0.193 | | None | |
| **Tamminen et al. (2020);** Spelling Task - Test 1 | 47 | Between-groups | Recall | Declarative | Yes | 15 | -0.108 | | None | |
| **Tamminen et al. (2020);** Phoneme Knowledge Task - Test 1 | 47 | Between-groups | Recall | Declarative | Yes | 15 | 0.005 | | None | |
| **Tamminen et al. (2020);** Old-New Decision Task - Test 2 | 46 | Between-groups | Recognition | Declarative | Yes | 15 | -0.330 | | None | |
| **Tamminen et al. (2020);** Reading Aloud Task - Test 2 | 46 | Between-groups | Recall | Declarative | Yes | 15 | -0.190 | | None | |
| **Tamminen et al. (2020);** Spelling Task - Test 2 | 47 | Between-groups | Recall | Declarative | Yes | 15 | -0.294 | | None | |
| **Tamminen et al. (2020);** Phoneme Knowledge Task - Test 2 | 47 | Between-groups | Recall | Declarative | Yes | 25 | -0.214 | | None | |
| **Kolibius et al. (2020);** Unpublished | 78 | Between-groups | Recall | Declarative | Yes | 23 | 0.249 | | None | |
| **Maddox et al. (2009)** | 43 | Between-groups | Categorisation | Declarative | No | 14 | 0.014 | | None | |
| **Albouy et al. (2013a)** | 31 | Between-groups | Motor Skill | Procedural | Yes | 12 | 2.445 | | None | |
| **Deliens et al. (2013b)** | 29 | Within-group | Recall | Declarative | Yes | 30 | -0.163 | | None | |
| **Herzog et al. (2012);** Serial Reaction Time Task; Low Energy Intake | 14 | Within-group | Recall | Procedural | No | 16 | 1.102 | | None | |
| **Herzog et al. (2012);** Serial Reaction Time Task; High Energy Intake | 14 | Within-group | Recall | Procedural | No | 16 | -0.118 | | None | |
| **Herzog et al. (2012);** Word Pair Recall Task; Low Energy Intake | 14 | Within-group | Recall | Declarative | No | 16 | 0.401 | | None | |
| **Herzog et al. (2012);** Word Pair Recall Task ; High Energy Intake | 14 | Within-group | Recall | Declarative | No | 16 | 0.550 | | None | |
| **Stickgold et al. (2000)** | 21 | Between-groups | Motor Skill | Procedural | Yes | 9 | 0.040 | | None | |
| **Ertelt et al. (2012);** Serial Reaction Time Task | 21 | Between-groups | Motor Skill | Procedural | Yes | 9 | 0.759 | | None | |
| **Ertelt et al. (2012);** Word Pair Associates Task | 21 | Between-groups | Recall | Declarative | Yes | 9 | -0.586 | | None | |
| **Rauchs et al. (2008);** Natural Condition -Day 4 | 24 | Between-groups | Recall | Declarative | Yes | 10 | 0.111 | | None | |
| **Rauchs et al. (2008);** Natural Condition - 6 Months | 23 | Between-groups | Recall | Declarative | Yes | 10 | 0.369 | | None | |
| **Rauchs et al. (2008);** Impoverished Condition - Day 4 | 24 | Between-groups | Recall | Declarative | Yes | 10 | 0.062 | | None | |
| **Rauchs et al. (2008);** Impoverished Condition - 6 Months | 23 | Between-groups | Recall | Declarative | Yes | 10 | -0.061 | | None | |
| **Rauchs et al. (2008);** Alternate Condition - Day 4 | 24 | Between-groups | Recall | Declarative | Yes | 10 | 0.053 | | None | |
| **Rauchs et al. (2008);** Alternate Condition - 6 Months | 23 | Between-groups | Recall | Declarative | Yes | 10 | 0.389 | | None | |
| **Rauchs et al. (2008);** Recognition Task - Day 4 | 24 | Between-groups | Recognition | Declarative | Yes | 10 | 0.118 | | None | |
| **Rauchs et al. (2008);** Recognition Task - 6 Months | 23 | Between-groups | Recognition | Declarative | Yes | 10 | 0.683 | | None | |
| ^1^ Citation specifies which effect size is referenced when multiple effect sizes could be extracted from a report. | | | | | | | |  | |  |
| ^2^ The moderator analysis included only studies using recall or recognition paradigms | | | | |  |  |  | |  | |
| ^3^ Power to detect the meta-analytic effect size | | | |  |  |  |  | |  | |
| *Meta-analysis moderators |  |  |  |  |  |  |  | |  | |

Appendix C

| Summary of studies investigating the effect of sleep deprivation before learning on memory | | | | |  |  | |  | |
| --- | --- | --- | --- | --- | --- | --- | --- | --- | --- |
| Citation^1^ | N | Study Design | Paradigm*^2^ | Memory Type* | Recovery sleep before test session?* | Power^3^* | | Effect Size | |
| **Basner et al. (2015);** Visual Object Learning Task | 44 | Within-group | Recognition | Declarative | No | 98 | | 0.178 | |
| **Bensimon et al. (1991);** Free Recall Task | 12 | Within-group | Recall | Declarative | No | 50 | | 1.148 | |
| **Boyle et al. (2012);** Delayed Word Recall Task | 16 | Within-group | Recall | Declarative | No | 64 | | -0.138 | |
| **Chatburn et al. (2017);** Verbal Recognition Memory | 22 | Within-group | Recognition | Declarative | No | 79 | | 0.643 | |
| **Chatburn et al. (2017);** Visual False Memory | 22 | Within-group | Recognition | Declarative | No | 79 | | 0.198 | |
| **Chee et al. (2010);** Attend Scenes Condition | 26 | Within-group | Recognition | Declarative | No | 86 | | -0.084 | |
| **Chee et al. (2010);** Attend Faces Condition | 26 | Within-group | Recognition | Declarative | No | 86 | | 0.169 | |
| **Chee et al. (2010);** Attend Faces & Scenes Condition - Face Score | 26 | Within-group | Recognition | Declarative | No | 86 | | 0.808 | |
| **Chee et al. (2010);** Attend Faces & Scenes Condition - Scene Score | 26 | Within-group | Recognition | Declarative | No | 86 | | 0.441 | |
| **Chuah et al. (2009)** | 26 | Within-group | Recognition | Declarative | No | 86 | | 0.776 | |
| **Drake et al. (2001);** Probed Memory Recall Task | 12 | Within-group | Recall | Declarative | No | 50 | | 0.307 | |
| **Drummond et al. (2000);** Free Recall Task | 13 | Within-group | Recall | Declarative | No | 53 | | 0.593 | |
| **Drummond et al. (2000);** Recognition Task | 13 | Within-group | Recognition | Declarative | No | 53 | | 0.468 | |
| **Drummond et al. (2005);** Free Recall Task | 32 | Within-group | Recall | Declarative | No | 53 | | 0.321 | |
| **Drummond et al. (2005);** Recognition Task | 32 | Within-group | Recognition | Declarative | No | 53 | | 0.424 | |
| **Esposito et al. (2015);** Prospective Memory Task | 50 | Between-groups | Recall | Declarative | No | 57 | | 1.087 | |
| **Falleti et al. (2003);** Associate Learning Task | 26 | Within-group | Recognition | Declarative | No | 86 | | 0.845 | |
| **Fischer et al. (2002);** Supplementary Experiment - Performance Rate | 16 | Between-groups | Finger Tapping | Procedural | No | 21 | | -0.034 | |
| **Forest et al. (2000);** Paired Word Recall Task - Semantically Associated Words | 18 | Between-groups | Recall | Declarative | No | 23 | | 1.264 | |
| **Forest et al. (2000);** Paired Word Recall Task - Semantically Unassociated Words | 18 | Between-groups | Recall | Declarative | No | 23 | | 1.260 | |
| **Giacobbo et al. (2016);** Free Recall Task | 39 | Between-groups | Recall | Declarative | No | 47 | | 0.975 | |
| **Grundgeiger et al. (2014);** Prospective Memory | 58 | Between-groups | Recall | Declarative | No | 64 | | 0.582 | |
| **Grundgeiger et al. (2014);** Recognition Memory | 58 | Between-groups | Recognition | Declarative | No | 64 | | 0.425 | |
| **Harrison et al. (2000a);** Temporal Recency Task | 20 | Between-groups | Recency Discrimination | Declarative | No | 26 | | 1.756 | |
| **Harrison et al. (2000a);** Temporal Recognition Task | 20 | Between-groups | Recognition | Declarative | No | 26 | | 0.957 | |
| **Harrison et al. (2000b);** Temporal Recency Task | 40 | Between-groups | Recency Discrimination | Declarative | No | 48 | | 0.507 | |
| **Harrison et al. (2000b);** Temporal Recognition Task | 40 | Between-groups | Recognition | Declarative | No | 48 | | -0.119 | |
| **Kaida et al. (2015);** Test 1 | 14 | Within-group | Recognition | Declarative | No | 58 | | 0.542 | |
| **Kaida et al. (2015);** Test 2 | 14 | Within-group | Recognition | Declarative | Yes | 58 | | 0.934 | |
| **Lo et al. (2016);** Experiment 1 | 38 | Between-groups | Recall | Declarative | No | 46 | | 0.192 | |
| **Maddox et al. (2011);** A/B Prototype Learning Task | 35 | Between-groups | Categorisation | Declarative | No | 43 | | 0.058 | |
| **Maddox et al. (2011);** A/Not-A Prototype Learning Task | 35 | Between-groups | Categorisation | Declarative | No | 43 | | 0.120 | |
| **Nilsson et al. (1989);** Cued Recall - Strong Pairs | 20 | Between-groups | Recall | Declarative | No | 26 | | 0.655 | |
| **Nilsson et al. (1989);** Cued Recall - Weak Pairs | 20 | Between-groups | Recall | Declarative | No | 26 | | 1.354 | |
| **Occhionero et al. (2017);** Interpolated Task | 50 | Between-groups | Recall | Declarative | No | 58 | | 0.345 | |
| **Occhionero et al. (2017);** Primary Task | 50 | Between-groups | Recall | Declarative | No | 58 | | 0.762 | |
| **Pace-Schott et al. (2009);** Temporal Memory Task | 25 | Between-groups | Recognition | Declarative | No | 32 | | 0.917 | |
| **Poh et al. (2017)** | 48 | Between-groups | Recognition | Declarative | No | 56 | | 1.426 | |
| **Roehrs et al. (2003);** Recall Task | 12 | Within-group | Recall | Declarative | No | 50 | | 0.649 | |
| **Stricker et al. (2006)** | 23 | Within-group | Recall | Declarative | No | 81 | | 0.503 | |
| **Tempesta et al. (2016);** Recognition Task | 48 | Between-groups | Recognition | Declarative | Yes | 56 | | 1.920 | |
| **Tempesta et al. (2016);** Temporal Order Task | 48 | Between-groups | Recognition | Declarative | Yes | 56 | | 0.990 | |
| **Yoo et al. (2007)** | 28 | Between-groups | Recognition | Declarative | Yes | 35 | | 2.142 | |
| **Tamminen et al. (2020);** Old-New Decision Task - Test 1 | 45 | Between-groups | Recognition | Declarative | Yes | 53 | | 0.324 | |
| **Tamminen et al. (2020);** Reading Aloud Task - Test 1 | 46 | Between-groups | Recall | Declarative | Yes | 55 | | 0.353 | |
| **Tamminen et al. (2020);** Spelling Task - Test 1 | 44 | Between-groups | Recall | Declarative | Yes | 52 | | 0.311 | |
| **Tamminen et al. (2020);** Phoneme Knowledge Task - Test 1 | 46 | Between-groups | Recall | Declarative | Yes | 54 | | 0.305 | |
| **Tamminen et al. (2020);** Old-New Decision Task - Test 2 | 45 | Between-groups | Recognition | Declarative | Yes | 53 | | 0.105 | |
| **Tamminen et al. (2020);** Reading Aloud Task - Test 2 | 46 | Between-groups | Recall | Declarative | Yes | 54 | | 0.235 | |
| **Tamminen et al. (2020);** Spelling Task - Test 2 | 44 | Between-groups | Recall | Declarative | Yes | 52 | | 0.250 | |
| **Tamminen et al. (2020);** Phoneme Knowledge Task - Test 2 | 46 | Between-groups | Recall | Declarative | Yes | 53 | | 0.384 | |
| **Blagrove et al. (1994);** Experiment 1; Day 2; Story Memory Task - Delayed Recall | 16 | Between-groups | Recall | Declarative | No | 21 | | 0.800 | |
| **Blagrove et al. (1994);** Experiment 2; Day 2; Story Memory Task - Delayed Recall | 24 | Between-groups | Recall | Declarative | No | 31 | | 1.322 | |
| **McWhirter et al. (2015)** | 18 | Between-groups | Texture Discrimination | Procedural | Yes | 24 | | 0.089 | |
| **Gorissen et al. (1997)** | 12 | Within-group | Recall | Declarative | No | 50 | | 0.326 | |
| ^1^ Citation specifies which effect size is referenced when multiple effect sizes could be extracted from a report.  ^2^ The moderator analysis included only studies using recall or recognition paradigms | | | | | | | | |  |
| ^3^ Power to detect the meta-analytic effect size | | | | | | |  |  |  |
| ^*^ Meta-analysis moderators | | | | | | |  |  |  |

Appendix D

Percentage of records that passed each item of the methodological quality checklist for each meta-analysis.

| Cluster | Item | Percentage of records that passed on each item | |
| --- | --- | --- | --- |
|  |  | Sleep Deprivation after Learning | Sleep Deprivation  before Learning |
| Reporting | 1. Neurological | 57.6 | 68.6 |
|  | 2. Psychiatric | 63.6 | 80.4 |
|  | 3. Sleep | 48.5 | 66.7 |
|  | 4. Medication | 37.4 | 39.2 |
|  | 5. Time Zones | 14.1 | 29.4 |
|  | 6. Chronotype | 36.4 | 56.9 |
| Internal Validity - Bias | 7. Pre-registered | 1 | 0 |
|  | 8. Optional Stopping | 9.1 | 19.6 |
|  | 9. Time of intervention and outcome the same? | 79.8 | 64.7 |
|  | 10. Pre-experimental Sleep | 62.6 | 82.4 |
|  | 11. Instructions | 89.9 | 82.4 |
|  | 12. Sleep Monitored | 87.9 | 74.5 |
|  | 13. Interference | 84.8 | 70.6 |
|  | 14. Day & Night Observation | 7.1 | 19.6 |
|  | 15. Night Observation | 89.9 | 94.1 |
| Internal Validity - Confounding | 16. Within-group | 20.2 | 39.2 |
|  | 17. Counterbalanced Conditions | 96 | 88.2 |
|  | 18. Random Allocation | 77.8 | 90.2 |
|  | 18. Similar Treatment | 100 | 100 |
|  | 20. Sleep Intertia | 76.8 | 76.5 |
|  | 21. Equal Sample | 90.9 | 94.1 |
| Power | 22. Power Analysis | 8.1 | 19.6 |
